# Engineered dCas9 with reduced toxicity in bacteria: implications for genetic circuit design

**DOI:** 10.1093/nar/gky884

**Published:** 2018-10-05

**Authors:** Shuyi Zhang, Christopher A Voigt

**Affiliations:** Department of Biological Engineering, Massachusetts Institute of Technology, Cambridge, MA 02139, USA

## Abstract

Large synthetic genetic circuits require the simultaneous expression of many regulators. Deactivated Cas9 (dCas9) can serve as a repressor by having a small guide RNA (sgRNA) direct it to bind a promoter. The programmability and specificity of RNA:DNA basepairing simplifies the generation of many orthogonal sgRNAs that, in theory, could serve as a large set of regulators in a circuit. However, dCas9 is toxic in many bacteria, thus limiting how high it can be expressed, and low concentrations are quickly sequestered by multiple sgRNAs. Here, we construct a non-toxic version of dCas9 by eliminating PAM (protospacer adjacent motif) binding with a R1335K mutation (dCas9*) and recovering DNA binding by fusing it to the PhlF repressor (dCas9*_PhlF). Both the 30 bp PhlF operator and 20 bp sgRNA binding site are required to repress a promoter. The larger region required for recognition mitigates toxicity in *Escherichia coli*, allowing up to 9600 ± 800 molecules of dCas9*_PhlF per cell before growth or morphology are impacted, as compared to 530 ± 40 molecules of dCas9. Further, PhlF multimerization leads to an increase in average cooperativity from *n* = 0.9 (dCas9) to 1.6 (dCas9*_PhlF). A set of 30 orthogonal sgRNA–promoter pairs are characterized as NOT gates; however, the simultaneous use of multiple sgRNAs leads to a monotonic decline in repression and after 15 are co-expressed the dynamic range is <10-fold. This work introduces a non-toxic variant of dCas9, critical for its use in applications in metabolic engineering and synthetic biology, and exposes a limitation in the number of regulators that can be used in one cell when they rely on a shared resource.

## INTRODUCTION

Synthetic regulatory networks enable the control of when genes are turned on (1). Natural networks can consist of hundreds of regulators, but implementing synthetic versions at this scale has proven elusive (2). Regulators used to build such networks have to perform reliably, cannot interfere with each other, and minimally tax cellular resources (3). Sets of protein-based repressors and activators have been used to build regulatory circuits, but expanding the set becomes increasingly difficult as each new protein needs to be tested for cross-reactions with the remainder in the set (4–8). Further, protein expression draws on cellular resources (ATP, ribosomes, amino acids, etc.), and this can result in slow growth, reduced metabolic performance, and evolutionary instability (9–11).

Regulators based on CRISPR (clustered regularly interspaced short palindromic repeats) machinery offer a potential solution (12–17). Catalytically inactive dCas9 can be used as a repressor by using the small guide RNA (sgRNA) to target a sequence within a promoter to sterically block RNA polymerase (RNAP) (18,19). The target sequence in the promoter is based on a 3 nt PAM sequence, which binds to the dCas9 protein, and a 20 nt targeting region that basepairs with the sgRNA. Different DNA sequences can be targeted by changing this region, which has been the basis for building large sets of sgRNA–promoter pairs that exhibit little or no crosstalk. Up to five pairs have been shown in *Escherichia coli* (20) and up to 20 pairs in yeast (21), but theoretically thousands could be made, essentially solving the need for orthogonal regulators to build large networks. In addition, sgRNA-circuits do not require translation to function, thus simplifying their use in the nucleus of eukaryotic cells. Previously, dCas9 has been used to build simple logic circuits and cascades with up to three sgRNAs in bacteria, seven sgRNAs in yeast and four sgRNAs in mammalian cells (20–26).

Despite the promise, there are several limitations in the scale-up of dCas9-based circuits. The foremost challenge is that high concentrations of dCas9 is toxic in many bacteria (27–29). This can be avoided for genome editing and CRISPR interference (CRISPRi) experiments by keeping the concentration low or limiting how long it is expressed (30). However, for a genetic circuit, dCas9 needs to be continuously available, including under the conditions required by the application, for example in a fermenter. This is compounded by the problem that multiple sgRNAs all have to share the same pool of dCas9. The draw-down of a shared resource leads to changes in performance of all the sgRNA, referred to as ‘retroactivity,’ and this can have a damaging impact on circuit function (31–34). Further, sgRNA-based gates have remarkably low cooperativity (Hill coefficient *n* ≈ 1.0) (20). Higher cooperativities are required to build regulation that implement multistable switches, feedback control, cascades, and oscillations (*n* > 1) (35–38). In yeast, the cooperativity of sgRNA-based regulation was increased by fusing dCas9 to the chromatin remodeling repression domain Mxi1, but there is no equivalent approach for prokaryotes (21).

The origins of dCas9 toxicity are poorly understood. It has been observed that dCas9 binds non-specifically to NGG PAM sites, particularly when unbound to a sgRNA, and there are many GG sequences in the genome (5.4 × 10^5^ PAM sites per *E. coli* genome) (39). While it primarily binds to this motif, it has been shown that it can also inefficiently recognize other PAM (e.g. NAG or NGA) sequences (40,41). Further, dCas9 functions by first actively interrogating the genome to search for the PAM motif, and then checking the complementarity of the sgRNA sequence to the target site (14,18). The search for PAM binding involves actively opening the DNA double strands in the chromosome (42). Previous studies also demonstrated that off-target genomic loci with up to six nucleotides that differ from the sgRNA sequence could still be recognized by Cas9, albeit with lower efficiency (but still requiring the PAM site) (43). These observations collectively point to the non-specific binding to NGG sequences by dCas9 as being a significant contributor to toxicity.

We hypothesized that reducing the non-specific binding of dCas9 would alleviate toxicity. The specificity of active Cas9 for genome editing applications has been increased via a variety of strategies, including point mutations to enhance PAM binding (44,45), increasing sgRNA length (46,47), splitting Cas9 (48–50), and the use of a pair of Cas9 nickases or FokI-dCas9 nucleases to increase the length of targeting sequence (51,52). It has been shown that Cas9 can be mutated (R1335K) to impair its ability to recognize the PAM, thus completely blocking DNA cleavage (53). Cleavage could be partially rescued by fusing a DNA binding protein (a ZFP or TALE) to dCas9 and placing the corresponding operator upstream of the region targeted by the sgRNA. The longer effective ‘operator’ increase cleavages specificity. Here, we apply this strategy to dCas9, but find that a fusion to the TetR-family PhlF repressor is uniquely able to recover full activity. We find this essentially eliminates toxicity, thus allowing up to 9600 proteins per cell without impairing cell health. Promoters are constructed that include the 30 bp PhlF operator and the sgRNA targeting sequence. A set of 30 sgRNAs are constructed and characterized as NOT gates with improved cooperativity (<*n>* = 1.6). Finally, we quantify the loss in dynamic range of a gate as additional sgRNAs are expressed and a mathematical model is used to quantify the loss in repression due to resource sharing. This work represents the first step towards harnessing dCas9 to scale-up circuit design; however, it also exposes limitations in the use of many regulators that require a shared pool of proteins for activity.

## MATERIALS AND METHODS

### Strains and media

All cloning was performed in *E. coli* NEB 10-beta (New England Biolabs, #C3019) and cells were grown in LB Miller broth (Difco, MI, #90003-350). The measurements experiments were done in *E. coli* K-12 MG1655 * [F-λ- ilvG- rfb-50 rph-1 Δ(araCBAD) Δ(LacI)] (20,54), and MOPS EZ Rich Defined Medium was used (Teknova, #M2105) with 0.2% glucose (Thermo Fisher Scientific, #156129) as carbon source for cell growth. Ampicillin (100 μg/ml, GoldBio, #A-301-5), kanamycin (50 μg/ml, GoldBio, #K-120-5), and spectinomycin sulfate (50 μg/ml, GoldBio, #S-140-5) were used to maintain plasmids when appropriate.

### Induction assays

Individual colonies were inoculated into 150 μl MOPS EZ Rich Defined Medium with appropriate antibiotics and then grown overnight (∼16 h) in 96-well plates (Nunc, Roskilde, Denmark, #249952) at 1000 rpm and 37°C on a plate shaker (ELMI, #DTS-4). Cultures were diluted 1000-fold by adding 2 μl of culture to 198 μl media, and then 15 μl of that dilution to 135 μl media, and grown with the same shaking condition for 3 h. At this point, cells were diluted 3000-fold by adding 2 μl of culture to 198 μl media, and then 5 μl of that dilution to 145 μl media with inducers and antibiotics as needed, and then were grown under the same conditions for 6 h.

### Flow cytometry analyses

Aliquots of 40 μl of media containing cells were collected and added to 160 μl phosphate-buffered saline with 1 mg/ml kanamycin to stop translation and arrest cell growth. The LSRII Fortessa flow cytometer (BD Biosciences, San Jose, CA, USA) was used to quantify the fluorescent protein production. The software FlowJo v10 (TreeStar, Inc., Ashland, OR) was used to gate the events by forward and side scatter, and at least 10 000 events were collected for each sample. The geometric mean of each sample was calculated. The autofluorescence of white cells was subtracted, defined as the geometric mean of a strain harboring an empty backbone (pSZ_Backbone, Supplementary Figure S12) grown under identical conditions. The fold-repression is measured as the uninduced divided by the induced fluorescence values. In Supplementary Figure S9, ±sgRNA fold-change indicates the repression of a promoter in the presence and absence of a plasmid from which the sgRNA is expressed. To calculate this, the fluorescence is measured for both strains and the ratio reported.

### Growth assay

Individual colonies were inoculated into 150 μl MOPS EZ Rich Defined Medium with appropriate antibiotics and then grown overnight (∼16 h) in 96-well plates (Nunc, Roskilde, Denmark, #249952) at 1000 rpm and 37°C on a plate shaker (ELMI, #DTS-4). Cultures were diluted 1000-fold by adding 2 μl of culture to 198 μl media, and then 15 μl of that dilution to 135 μl media, and grown with the same shaking condition for 3 h. After the 3 h step, the cultures were diluted 3000-fold by adding 2 μl of culture to 198 μl media, and then 5 μl of that dilution to 145 μl media with appropriate antibiotics and different inducers concentrations. The dilutions were made in 96-well plates (Nunc, Roskilde, Denmark, #165305) and grown at 1000 rpm and 37°C for 6 h. The optical density at 600 nm was measured on a Synergy H1 plate reader (BioTek, Winooski, VT, USA) and the background of MOPS EZ Rich Defined Medium was subtracted. The measured values were then normalized to the un-induced samples (0 ng/ml aTc).

### Microscopy

During the growth assay, after 6 hours, aliquots (2 μl) of cultures were collected. Microscopic images of these cultures were then taken on the Axiovert 200m microscope (Carl Zeiss, Oberkochen, Germany).

### Numbers of cells per ml

Colonies were inoculated into 150 μl MOPS EZ Rich Defined Medium with appropriate antibiotics and then grown overnight (∼16 h). The next day, these cultures were diluted by adding 1 μl culture into 1 ml fresh media. After 5 h of growth (1000 rpm and 37°C), the culture density was measured and diluted to different OD_600nm_. The cultures at different OD_600nm_ were then diluted 2 × 10^7^-fold and plated on LB agar. Colony numbers were then counted after overnight growth at 37°C.

### Quantification of dCas9

Colonies were inoculated into 150 μl MOPS EZ Rich Defined Medium with appropriate antibiotics and then grown overnight (∼16 h). The next day, these cultures were diluted by adding 1 μl culture into 1 ml fresh media containing inducer (2.5 ng/ml or 0.7 ng/ml aTc). After 5 h of growth (1000 rpm and 37°C), the culture density was measured and adjusted to OD_600nm_ = 1 with MOPS EZ Rich Defined Medium. 700 μl of the adjusted culture for each strain was centrifuged at 12 000 rpm for 1 min. The supernatant was discarded and cell pellet was re-suspended in 40 μl lysis buffer (100 mM NaCl, 25 mM Tris–HCl, pH 8.0) containing 0.2 % β-mercaptoethanol (Sigma-Aldrich, #M6250). The samples were boiled at 100°C for 5 min, after which 3 μl of the dCas9 sample and 0.75 μl of the dCas9*_PhlF sample were added to lysis buffer to a final volume of 20 μl.

To prepare the standard curve, 2 μl of purchased Cas9 complex (New England Biolabs, #M0386S) was added to 38 μl lysis buffer. Then, different amounts (0.2, 1, 3, 5 μl) of the diluted Cas9 standard, 3 μl WT lysate, and lysis buffer were added to each sample to a total volume of 20 μl.

The same amount (10 μl) of the resulting standards and cell lysates were loaded on a 4–12 % gradient SDS-PAGE gel (Lonza, # 59524). After the run, the gels were transferred onto a PVDF membrane (Biorad, #162-0177) and then blocked at room temperature for 1 h in 5% skim milk (w/v of TBST, 138 mM NaCl, 2.7 mM KCl, 0.1% Triton X-100, 25 mM Tris–HCl, pH 8.0). The anti-Cas9 antibody (abcam, #ab202580) was used as primary antibody and added 1:2000 into 2.5% skim milk (w/v of TBST). The primary antibody solution was then added to the PVDF membrane and allowed to bind for 1 hour at room temperature. The membrane was then washed three times with TBST. The secondary antibody, HRP-conjugated anti-mouse antibody (Sigma, #A8924), was added to 1:4000 and incubated for 1 h at room temperature. After washing the membrane, chemiluminescence for HRP (Pierce, #32106) was used to develop the signal and detected using the Biorad chemidoc MP imaging system (Biorad, #170-8280). ImageJ 1.41 (NIH) was used to analyze the gel densitometry. The relative protein numbers of dCas9 in the strain was calculated from the standard curve and known concentrations of Cas9 standards (Supplementary Figure S3).

### Random sequence generation

The random sequences are generated using the online Random DNA Sequence Generator (http://www.faculty.ucr.edu/∼mmaduro/random.htm) with GC content set to 50%.

### sgRNA array

Pairs of ssDNA oligonucleotides ≤200 nt long that encode the necessary genetic parts (promoter, sgRNA, terminator) were ordered from Integrated DNA Technologies (IDT). These oligos are annealed by PCR using KAPA HiFi MasterMix (KAPA Biosystems, #07958935001) and the resulting dsDNA modules were then assembled in a one-pot Golden Gate assembly reaction using type II enzymes BsaI (New England Biolabs, #R0535S) or BsmbI (New England Biolabs, #R0580S) to generate plasmids with different numbers of sgRNAs. After transformation, these plasmids were re-purified and digested with restriction enzyme BsphI (New England Biolabs, #R0517S) to make sure they have the expected sizes and thus rule out the possibility of unwanted homologous recombination during construction and transformation (Supplementary Figure S8).

### Energy cost of expressing dCas9*_PhlF and TetR

The following is an estimation of the number of ATPs required to synthesize a TetR repressor and dCas9*_PhlF. The *tetR* gene is 624 bp and the TetR protein contains 207 amino acids. The cost of mRNA has been estimated to be 6 ATP per triplet (55), making the total cost of the tetR mRNA to be approximately 1242 ATP. On average one mRNA produces 30 proteins, thus the cost per protein is 41 ATP (55). Using Table 1 from ref. (55), the cost of making the amino acids for TetR is -307 ATP (negative indicating a net increase of ATP). Translation costs 4 ATP/amino acid. Taking all of these factors into account the total cost of one TetR protein is 562 ATP. Following a similar calculation, the cost of dCas9*_PhlF is 5551 ATP.

## RESULTS

### Design and testing of a dCas9 variant with reduced toxicity

**Figure 1.**
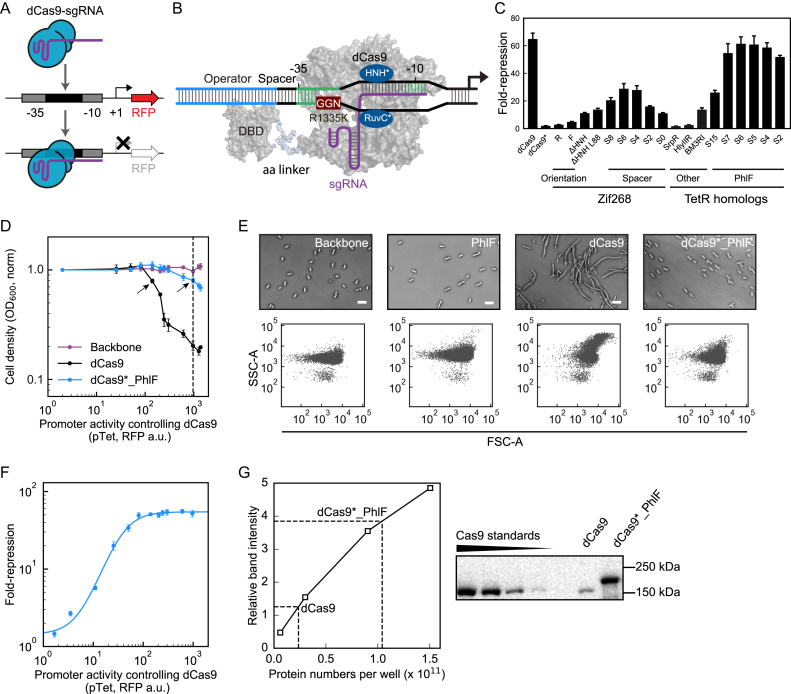
Design and evaluation of a dCas9 – transcription factor fusion. (**A**) A schematic of targeted repression by dCas9-sgRNA complex bound to the promoter region of a fluorescent reporter gene (RFP, red fluorescent protein). (**B**) A detailed schematic of the fused protein bound to a promoter is shown. DBD is the DNA-binding domain that is fused to dCas9. GGN is the PAM site. R1335K is the mutation that reduces the PAM recognition ability of dCas9. (**C**) The impact of changes to the fused protein and promoter on the response. The fold-repression is calculated as the ratio of uninduced to induced (1 mM IPTG) cells (Methods). All constructs other than the first are based on dCas9* (R1335K). F and R represent the forward and reverse orientations of the Zif268 operator. ΔHNH refers to the deletion of this domain. L88 shows the impact of a longer linker. The size of the spacer between the -35 and operator sequence is shown as S*N*, where *N* is the number of bp. Sequences and plasmid maps are shown in Supplementary Figure S12 and Supplementary Table S2. SrpR, HlyIIR and BM3RI are all TetR-family repressors that were tested as alternatives to PhlF. (**D**) The growth impact of dCas9 and dCas9*_PhlF is compared to the pSZ_Backbone plasmid (Supplementary Figure S12) as a control. Protein expression is controlled using the aTc-inducible system and the x-axis is shown in units of fluorescence for the pTet promoter, measured separately (Supplementary Figure S1). The dashed line shows 2.5 ng/ml aTc, used in E for morphology studies. The arrows point to the inducer levels (0.7 ng/ml and 2.5 ng/ml) where the protein concentrations are determined in Figure 1G. Media and growth conditions are provided in the Materials and Methods. (**E**) Microscopic images of *E. coli* strains expressing PhlF, dCas9 or dCas9*_PhlF variants and a control (Backbone) are shown, under identical conditions as used for the growth curves. The scale bars are 5 μm. The corresponding FSC-A/SSC-A distribution of each strain was measured by flow cytometry (Materials and Methods). (**F**) The fold-repression of the construct (pSZ_PhlF plasmid in Supplementary Figure S12 and the pPhlF_S6 promoter from Supplementary Table S2) is shown as a function of dCas9*_PhlF expression. The sgRNA is under the control of the pTac promoter and all data are for 1 mM IPTG. The x-axis is the same as described in D. The line shows a fit to a Hill equation. For B–F, the data are shown as the mean of three experiments performed on different days and the error bars are the standard deviation. (**G**) A representative immunoblotting assay is shown for calculating the number of dCas9 per cell. The dashed lines show the interpolation used to estimate concentrations. The calculation is described in the Methods and the numbers presented in the text are based on three experiments performed on different days (Supplementary Figure S3).

Transcription of a target reporter gene is blocked when a dCas9-sgRNA complex binds to its promoter (Figure 1A). Following the hypothesis that non-specific dCas9 binding to DNA leads to its toxicity, we made a series of mutations intended to disrupt binding. A schematic of these modifications is shown in Figure 1B. The RuvC* and HNH* domains are mutated to disrupt the nuclease activity of Cas9 to create dCas9 (18). The promoter is based on the strong constitutive promoter BBa_J23101 (20), modified upstream of the −10 position to contain a 20 bp sequence that is complementary to the cognate sgRNA. The activity of this promoter is measured using a transcriptional fusion to red fluorescent protein (*rfp*) and flow cytometry (Materials and Methods). Binding to the PAM site (NGG) is disrupted by making the R1335K mutation to dCas9 (53). Various DNA-binding domains (DBD) are fused to the C-terminal end of dCas9 and the corresponding operator is placed upstream of the −35 promoter region, separated by a spacer. A linker is used to control the distance between the DBD and dCas9.

A reporter system was developed to evaluate the impact of these modifications on the ability for dCas9 to repress the targeted promoter (Supplementary Figure S12). The expression of the sgRNA and dCas9 are controlled using IPTG- and aTc- inducible promoters, respectively. All of these components are integrated into a single p15A plasmid backbone. The fold-repression is measured as the fluorescence from the output promoter in the absence of sgRNA inducer (0 mM IPTG), divided by the fluorescence when the sgRNA is expressed (1 mM IPTG). When the R1335K mutation is made (dCas9*), this completely abolishes repression as expected (Figure 1C).

We first tested the ability for a zinc finger protein (ZFP) to recover nuclease activity. To this end, we built a variant of dCas9* described previously, where Zif268^TS3^ is fused to the C-terminal end of dCas9* via a 58 amino acid linker (53). The corresponding 12 bp operator recognized by Zif268^TS3^ was then placed upstream of the promoter, separated from the −35 position by a spacer (all promoter variants described are provided in Supplementary Table S2). The orientation of the operator (forward and reverse) was initially tested with the forward yielding higher repression as previously observed (53). Thus, it was selected for all subsequent optimization. The deletion of the nuclease domain (ΔHNH) (56) and the increase in linker size to 88 amino acids (L88) both improved repression (Figure 1C). Finally, the length of the spacer was varied between 0 to 8 bp and an optimum was identified at 6. Collectively, these changes resulted in a ZFP fused dCas9 that can only achieve a maximum of 28-fold repression, roughly a third of the activity of the unmodified variant.

TetR-family repressors were then evaluated in place of the ZFP using the same dCas9* variant (88 amino acid linker, ΔHNH). Four repressors were tested (PhlF, BM3RI, HlyIIR, and SrpR) and their corresponding operators (30, 20, 22, 30 bp, Supplementary Table S2) were inserted in front of the promoter with the 6 bp spacer (8). Of these, the PhlF fusion (dCas9*_PhlF) recovered the most activity, achieving 95% of the repression of dCas9 with an optimal spacer length of 6 bp (Figure 1C).

The growth impact of dCas9 was then compared to dCas9*_PhlF at different levels of expression, controlled by the addition of aTc. The activity of the pTet promoter is used as a surrogate of dCas9 expression, measured in independent experiments using a separate plasmid and red fluorescent protein (Supplementary Figure S1). There is a clear impact on growth, where cells expressing dCas9 rapidly declines past an expression threshold (Figure 1D). In contrast, there is only a slight defect at the highest expression levels of dCas9*_PhlF. The morphological impact on the cell can be seen when aliquots are compared at the same level of inducer (2.5 ng/ml aTc) (Figure 1E). The expression of dCas9* leads to longer cells and larger side scatter (SSC-A) (57), an effect described previously (28). However, when expressing dCas9*_PhlF or PhlF alone, the same level of inducer leads to cell morphologies similar to wild-type *E. coli*. Next, we tested whether the changes made to build dCas9*_PhlF simply disrupted its ability to act as a repressor. Repression saturates at an expression level well before any growth defect is observed (Figure 1F), thus indicating the changes are not impacting performance.

Note that the use of promoter strengths to compare expression levels between dCas9 and dCas9*_PhlF is, at best, inexact as these genes will translate differently. Therefore, we performed immunoblotting to quantify the size of the pools of each protein that the cell can tolerate before a growth impact is observed. Based on the growth experiment, we chose 0.7 ng/ml aTc for dCas9 and 2.5 ng/ml aTc for dCas9*_PhlF as the maximum inducer levels before growth is impacted. (arrows in Figure 1D). The details of these experiments are presented in the Materials and Methods. Briefly, a standard curve was generated using commercially-available Cas9 of known concentration and a Cas9-targeting monoclonal antibody (Figure 1G). Then, wells are loaded with whole cell lysate from strains expressing dCas9 or dCas9*_PhlF and the dCas9 number per well is calculated from band intensity of that well by comparing to the standard curve. The number of cells per ml were also measured and used in the calculation (Supplementary Figure S2). The average of three biological replicates, one of which is shown in Figure 1G, determined that 9600 ± 800 molecules of dCas9*_PhlF and 530 ± 40 molecules of dCas9* are tolerated by a cell before growth and morphology defects are observed (Supplementary Figure S3).

### Design and characterization of sgRNA–promoter pairs as NOT gates

**Figure 2.**
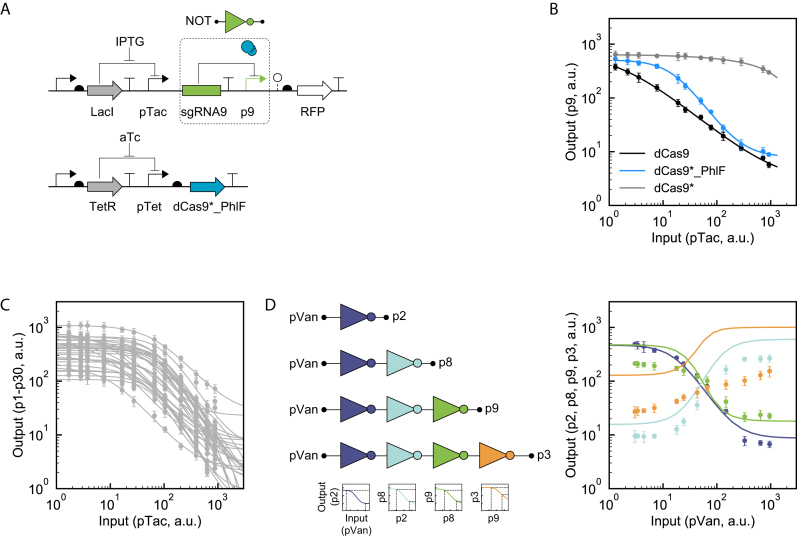
NOT gates based on dCas9*_PhlF. (**A**) The schematic of the gate is shown. The input and output to the gate are pTac and p9. Part sequences and plasmid maps are provided in Supplementary Figure S12 and Supplementary Table S3. (**B**) The response curves of dCas9-based NOT gates are shown (Methods). The input is the activity of the pTac promoter as a function of IPTG concentration, measured separately (Supplementary Figure S1). The concentration of dCas9*_PhlF was maintained by adding 2.5 ng/ml aTc and 0.7 ng/ml for dCas9. (**C**) The response functions of 30 NOT gated based on orthogonal pairs of sgRNAs and promoters. The sequences are provided in Supplementary Figure S11. The data were fit to Equation (1) and the resulting parameters are provided in Supplementary Table S1. (**D**) Evaluation of cascades of different length. The detailed parts used in the genetic systems are shown in Supplementary Figure S13. The color of the gate indicates the sgRNA:promoter pair used (blue: sgRNA2, light blue: sgRNA8, green: sgRNA9, orange: sgRNA3). The input to the gate is the vanillic acid inducible promoter (pVan) and the x-axis is the activity of this promoter at different levels of inducer, measured separately (Supplementary Figure S1). The color of the data corresponds to the last gate of the cascade. The fits to the data are the responses predicted by combining the response functions of each layer of the cascade. The response functions of the individual gates and the predicted propagation of the signal through the cascade are shown at the bottom (Methods). All of the data in this Figure are shown as the mean of three experiments performed on different days and the error bars are the standard deviation.

A transcriptional NOT gate inverts the response of a promoter (58). More complex circuits can be constructed by connecting NOT gates to each other (e.g. toggle switch and oscillator) or by converting to NOR gates through the addition of a second upstream input promoter (7,38,59,60). Previously, we designed an architecture for NOT and NOR gates based on sgRNAs using dCas9 (20). Here, we followed this approach to build gates based on dCas9*_PhlF, where the input promoter driving sgRNA is an IPTG-inducible pTac promoter (Figure 2A). The response of the output promoter is measured using a transcriptional fusion to *rfp*. These were combined to build a single plasmid using the p15A backbone (Supplementary Figure S12). The plasmid was transformed into *E. coli* and cells were grown in inducer until reaching steady-state (Materials and Methods).

The response function is characterized by comparing the activity of the pTac promoter, measured separately, versus the activity of the output promoter (Figure 2B and Materials and Methods). The resulting data can be fit to the equation,

(1) \begin{equation*}y\ = {Y_{min}}\ + \left( {{Y_{max}} - \ {Y_{min}}} \right)\frac{{{K^n}}}{{{x^n} + {K^n}}},\end{equation*}

where *y* is the output promoter activity (and *Y_max_/Y_min_* are the maximum/minimum activities), *x* is the input promoter activity, *K* is the threshold and *n* is the cooperativity. Note that the values of the promoter activities are in arbitrary units of red fluorescence and not standardized units. The response function from dCas9 is linear over the entire range of input with *n* = 0.9, as observed previously (Figure 2B). However, the response function resulting from dCas9*_PhlF has a clear S-shape with *n* = 1.6. The increased cooperativity could be due to the multimerization of PhlF, a mechanism supported by the loss in repression observed by adding the PhlF inducer DAPG (Supplementary Figure S4).

A library of NOT gates was then built based on a set of 30 orthogonal sgRNAs (20). The target sequence corresponding to each was used to construct a promoter based on the system shown in Figure 1B. The resulting NOT gates were then characterized as before and fit to Equation (1). The shapes of the curves are similar, but the maximum activity shifts as a result of the operator changes impacting promoter strength (Figure 2C). On average, the gates exhibit a 47-fold dynamic range and the cooperativities span from 1.3 to 1.8. Because there are no cross reactions between gates, these could be used as the basis for the construction of large genetic circuits.

Cascades were constructed to demonstrate the layering of gates. First, the vanillic acid inducible system (pVan) was selected to serve as the input because it was observed to generate the largest dynamic range (341-fold) (Supplementary Figure S5). This was used as the input for a series of cascades based on 1 to 4 sgRNAs (Figure 2D and Supplementary Figure S6). The predicted response (solid lines) of each cascade was calculated by mathematically combining the response functions of the individually-measured gates. For the first three layers, the measured response closely matches that predicted. However, the addition of the fourth layer leads to a significant deviation from the predicted response. When dCas9*_PhlF was expressed at lower levels, the measured responses deviated from the predicted responses even in the first two layers (Supplementary Figure S7).

### Sharing of the dCas9*_PhlF pool by multiple sgRNAs

Genetic circuits with more than one gate require the simultaneous expression of multiple sgRNAs within the cell that need to compete with the same pool of dCas9 molecules. The sharing impacts the dynamics of each component in the system and this can have unintended consequences for the overall behavior of the circuit (31). Therefore, it is important to quantify the titration that occurs as more sgRNAs are simultaneously expressed.

**Figure 3.**
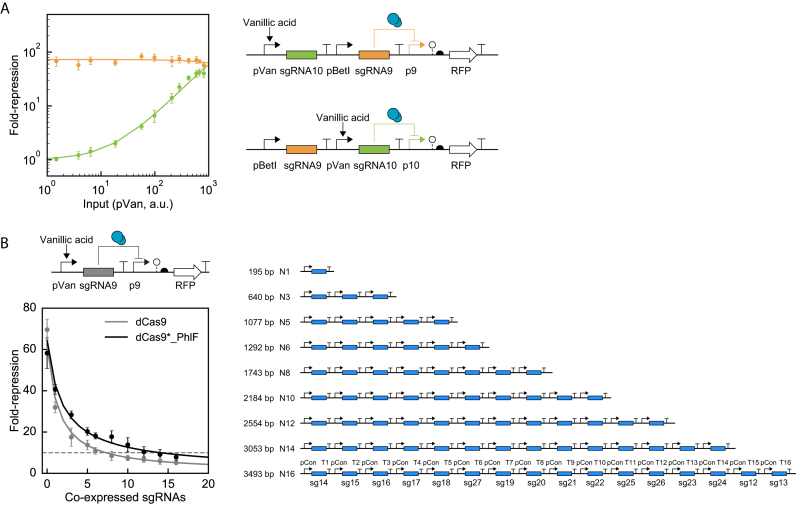
The impact of simultaneous expression of multiple sgRNAs. (**A**) Expression of sgRNA9 was fully induced (10 mM choline, activating pBetI) to measure fold-repression of promoter p9 (orange), while the expression level of sgRNA10 (green) was induced by adding different levels of vanillic acid. The activity of the pVan promoter was measured separately as a function of vanillic acid concentration (Supplementary Figure S1). The detailed parts used in the genetic systems are shown in Supplementary Figure S13. Solid lines are model prediction results. (**B**) The impact of expressing multiple sgRNAs simultaneously. The repression fold change of promoter p9 was measured with or without the addition 100 μM vanillic acid. The constructs containing different numbers of sgRNAs are shown to the right. The sequences corresponding to the promoters and terminators are provided in Supplementary Table S3. The sgRNAs are labeled sg*N* where *N* corresponds to the sequences in Supplementary Figure S11. The horizontal line marks 10-fold repression, roughly the minimum required for useful NOT gates. For dCas9*_PhlF, the fit parameters for Equations (11) and (12) are *β* = 3.0 × 10^−11^ Ms^−1^, *α_1_* = 7.6 × 10^−12^ Ms^−1^, *α_x_* = 2.3 × 10^−11^ Ms^−1^, *K* = 1.7 × 10^−8^ M, *n* = 0.9. For dCas9, the fit parameters are: *β* = 3.0 × 10^−11^ Ms^−1^, *α_1_* = 7.6 × 10^−12^ Ms^−1^, *α_x_* = 2.3 × 10^−11^ Ms^−1^, *K* = 2.9 × 10^−9^ M, *n* = 1.1, In both parts, the data are shown as the mean of three experiments performed on different days and the error bars are the standard deviation.

First, we characterized the impact of resource sharing between two sgRNAs (Figure 3A). The pBetI promoter was used to generate a constitutive level of sgRNA9, which represses the p9 promoter. The vanillic acid inducible promoter (pVan) then drives a second sgRNA10. As vanillic acid is added and the second sgRNA is transcribed at higher levels, there is almost no impact on the ability of the first to repress its promoter. This is true even when sgRNA10 is expressed at the level required for the full repression of its cognate p10 promoter. Therefore, both sgRNAs can be fully expressed and independently repress two promoters without incurring significant effects due to resource sharing.

It is expected that as more sgRNAs are added to the system, at some point there would be a decline in their ability to function as dCas9*_PhlF is titrated. To quantify this transition, we developed a mathematical model inspired closely by the work of Del Vecchio and co-workers (BioRxiv: https://www.biorxiv.org/content/early/2018/02/14/266015). The equations corresponding to when two sgRNAs are expressed are described below and this is expanded to a system of *i* sgRNAs in the Supplementary Note. The pool of total dCas9 *C_TOT_* is assumed to be constant. It can be described as the algebraic sum of free dCas9 *C_F_* and the concentrations of dCas9 bound to the first and second sgRNAs (*s*_1_ and *s*_2_),

(2) \begin{equation*}{C_{TOT}} = {C_F}\ + {C_{s1}} + {C_{s2}}\end{equation*}

The dynamics of the unbound sgRNAs *s*_1_ and *s*_2_ are captured by the differential equations

(3) \begin{equation*}\frac{{d{s_1}}}{{dt}} = {\alpha _1}\ - {\delta _s}{s_1} - {k_1}{C_F}{s_1} + {k_{ - 1}}{C_{s1}}\qquad {\rm{and}}\end{equation*}

(4) \begin{equation*}\frac{{d{s_2}}}{{dt}} = {\alpha _2}\ - {\delta _s}{s_2} - {k_1}{C_F}{s_2} + {k_{ - 1}}{C_{s2}}\qquad {\rm{and}}\end{equation*}

where *α_1_* and *α_2_* are the transcription rates of the first and second sgRNAs. *δ_s_* is degradation rates, and assumed to be the same for different sgRNAs. Similarly, the on- and off-rates of sgRNAs to dCas9 (*k*_1_ and *k*_-1_) are assumed to be sequence independent. There are two additional differential equations for the formation of sgRNA::dCas9 complexes:

(5) \begin{equation*}\frac{{d{C_{s1}}}}{{dt}} = {k_1}{C_F}\ {s_1} - {k_{ - 1}}{C_{s1}}\qquad {\rm{and}}\end{equation*}

(6) \begin{equation*}\frac{{d{C_{s2}}}}{{dt}} = {k_1}{C_F}\ {s_2} - {k_{ - 1}}{C_{s2}}\ \end{equation*}

Finally, the concentration of free dCas9 is given by

(7) \begin{equation*}\frac{{d{C_F}}}{{dt}} = - {k_1}{C_F}\ {s_1} - {k_1}{C_F}{s_2} + {k_{ - 1}}{C_{s1}} + {k_{ - 1}}{C_{s2}}.\ \end{equation*}

At steady-state, Equations (2–7) reduce to

(8) \begin{equation*}{s_1} = \frac{{{\alpha _1}}}{{{\delta _s}}}\qquad {\rm{and}}\end{equation*}

(9) \begin{equation*}{C_{s1}} = \frac{{{K_1}{s_1}{C_{TOT}}}}{{1 + {K_1}{s_1} + {K_1}{s_2}}},\end{equation*}

where *K_1_* is the association equilibrium constant of sgRNA to dCas9. This captures how increasing the concentration of the second sgRNA impacts the concentration of complexes with the first. By substituting sgRNA concentration from Equation (8), we can simplify Equation (9) to

(10) \begin{equation*}{C_{s1}} = \frac{{{\alpha _1}{C_{TOT}}}}{{\beta + {\alpha _1} + {\alpha _2}}},\ \end{equation*}

where $\beta \ = \ \frac{{{\delta _s}}}{{{K_1}}}$.

Considering a Shea–Acker's model of a repressor binding to a promoter (related in form to Equation 1), the impact on transcription would be:

(11) \begin{equation*}\frac{G}{{{G_{ss}}}} = \ 1 + \frac{{{C_{s1}}^n}}{{{K^n}}},\end{equation*}

where *G/G_ss_* is the fold-repression, *K* is the dissociation equilibrium constant for dCas9::sgRNA binding to the promoter, and *n* is the cooperativity. Combining Equations (10) and (11) shows how the expression of a second sgRNA impact the repression of promoter responsive to the first sgRNA.

Similarly, concentration of the first sgRNA::dCas9 complex can be derived when multiple competing sgRNAs are co-expressed and sharing the dCas9 pool (Supplementary note):

(12) \begin{equation*}{C_{s1}} = \frac{{{\alpha _1}{C_{TOT}}}}{{\beta + {\alpha _1} + N{\alpha _X}}}\end{equation*}

where *N* is the number of additional co-expressed sgRNAs and *α_x_* is the transcription rate of these competing sgRNAs. The concentration for each of these competing sgRNAs is assumed to be equal. The fold-repression is calculated by substituting *C_s1_* from Equation (12) into Equation (11).

To parameterize the model, we measured how the response of a sgRNA declines as more competing sgRNAs are added to the system. The response of a vanillic acid-driven NOT gate based on sgRNA9 was measured; alone, it generates 58-fold repression (Figure 3B). Then, a series of constructs were designed to express increasing numbers of sgRNAs, from 1 to 16. Each expression unit consists of the same pCon constitutive promoter, a different sgRNA (but conserving the tracrRNA sequence), and different strong terminators (part sequences in Supplementary Table S3). The constructs involve the repetition of these units within a single construct. While effort was made to minimize repetitive DNA, sufficient regions of sequence similarity remain so special cloning procedures were used and construct stability confirmed by digestion (Methods and Supplementary Figure S8).

Our goal was to evaluate a maximum number of sgRNAs that can be used together. Therefore, we tuned the system to minimize the expression level of each sgRNA to the point where they are as low as possible but still could minimally function as a NOT gate. In accordance with this approach, the constitutive promoter (pCon) was selected such that each sgRNA yields ∼10-fold repression when measured in the context of the N16 construct (Supplementary Figure S9). In essence, this maximizes the number of sgRNAs that can be used simultaneously, thus representing an upper limit. The growth impact of expressing sgRNAs was also measured and only a slight decrease in the growth of *E. coli* cell was observed as more sgRNAs were simultaneously expressed (Supplementary Figure S10).

The impact on the sgRNA9 gate was measured as a function of the number of additional sgRNAs co-expressed (Figure 3B). The additional sgRNAs do not bind to any DNA sequences in the system because their cognate promoters are not included. This response was compared for both dCas9 and dCas9*_PhlF expressed to the maximal level prior to observing a growth defect (0.7 and 2.5 ng/ml aTc, respectively). In both cases, there is a significant decline in repression even with the first few additional sgRNAs. The slope is steeper for dCas9 and the response falls below 10-fold after seven more sgRNAs are co-expressed, while for dCas9*_PhlF this increases to 14 sgRNAs.

## DISCUSSION

The original uses intended for Cas9 and dCas9 have different constraints than those required for genetic circuits. Genome editing and knockdown experiments only require transient and low levels of expression for activity. These applications benefit from the capability of sgRNA to be designed to target essentially any region of the genome and this programmability could be very useful for building out sets of orthogonal regulators for genetic circuits. However, integrating a circuit into an application is more complicated, for example to produce a chemical product in a fermenter or integrate information in the human gut (61–65). For these purposes, a circuit cannot reduce growth or require significant cellular resources or energy to function. In this manuscript, we have solved one of these problems, where the growth impact of dCas9 is greatly reduced by increasing the required DNA sequence to which it binds by swapping a 3 bp PAM site for a 30 bp PhlF operator. This allows the expression of dCas9*_PhlF to be increased to ∼10^4^ copies per cell, which is just about as high as one can expect to push the expression of a large protein in *E. coli* (66).

Repetitive sequences shared between gates is another challenge that must be solved before large sgRNA circuits can be built based on dCas9*_PhlF. The shared sequences can lead to genetic instability due to homologous recombination (67,68). All of the sgRNA-based gates share the identical 83 bp tracrRNA sequences, and the output promoters share the identical 30 bp PhlF operator (Supplementary Figure S11). In addition, converting the NOT gates to NOR gates requires either duplicating the sgRNA or using a ribozyme to cleave 5′-UTR generated by two upstream promoters in series (7,20,21). Both of these approaches lead to longer regions of repeated DNA. Stabilizing circuits would require sequence diversification and the creation of part libraries (e.g. of ribozymes) with diverse sequences, approaches that have been applied previously (69,70).

However, before undertaking this effort, it is important to consider whether the concept makes sense. The pool of dCas9*_PhlF would need to be maintained at a constant ∼10^4^ molecules irrespective of the number of active gates. Our experimental data and model show that this can support about 15 sgRNA-based gates (Supplementary note). This is about on par with the number of available protein-based gates and is a harsh limitation to the huge number of potential gates considering sgRNA programmability alone (estimated to be ∼10^7^ sgRNA–promoter pairs) (20). The retroactivity due to having to share the dCas9*_PhlF resource also changes as each additional sgRNAs is added to the system. When designing circuits, a mathematical model would have to be used to mitigate this complexity. Thus, the benefit of sgRNA-based gates, even when the dCas9 toxicity is solved, is not a dramatic increase in circuit size. However, there may be other benefits in some cases.

One such scenario may be in eukaryotes where using dCas9-based gates have an advantage (6,21,71). The lack of translation at the gate level means that that circuit function can be entirely localized to the nucleus (once a dCas9 pool has been imported), thus avoiding the problem with protein-based gates, where the regulator mRNA must be exported from the nucleus and the regulator protein imported. Another may be for organisms where for which the circuit needs to be carried at low copy and the design of high-expression promoters remains elusive (63).

It is not true that sgRNA gates require less cellular resources because they do not require translation to function. While each gate only requires a new sgRNA to be transcribed, for it to be functional it needs a dCas9*_PhlF to form a complex that represses the output promoter. The binding of sgRNA to dCas9 is very tight (*K*_d_ = 10 pM) (50) and dCas9 binds tightly to DNA (*K*_d_ = 1 nM) (42,72,73), requiring DNA replication machinery for removal during division (39). Therefore, it is likely that recycling of the pool will be low (reuse of dCas9 after dissociating from a previous sgRNA). This makes the cost of each dCas9*_PhlF:sgRNA ‘repressor’ high when compared to a protein-based repressor (e.g. TetR). Putting it in terms of ATP consumption, an estimation is that the former requires ∼5000 ATP/repressor and the latter ∼500 ATP/repressor (Methods).

The sharing of a resource is a common feature of cells, including natural regulatory networks (74,75). One example are sigma factors, turned on in response to different cellular needs, that all must share core RNA polymerase to initiate transcription from a promoter (76). If multiple sigma factors were co-expressed, this would draw down the core resource. It has been shown that *B. subtilis* has an innovative solution: each sigma factor is expressed as an independent pulse and the pulsing time is changed with respect to need, as opposed to the expression level (77). In the natural network, this is achieved with feedback loops of a complexity still difficult to achieve in engineered systems. Our results point to the difficulty of using a genetic circuit paradigm that requires a shared (and expensive) non-recyclable resource in bacteria. This work highlights the need to develop theoretical and experimental frameworks to quantify the cellular impact of introducing systems into cells, prior to performing experiments, in order to rationally guide design decisions.

## Supplementary Material

Supplementary Data
