# Supplementary material for: Engineered dCas9 with reduced toxicity in bacteria: implications for genetic circuit design
Source: Nucleic Acids Res. 2018 Oct 5;46(20):11115–25. doi: 10.1093/nar/gky884 (PMC6237744; doi:10.1093/nar/gky884)
Supplement: Supplementary Data [file gky884_supplemental_files.docx]

Supplementary Information for:

**Engineered dCas9 with reduced toxicity in bacteria: implications for genetic circuit design**

Shuyi Zhang and Christopher A. Voigt

[Supplementary Note: Derivation for the impact of dCas9 sharing by multiple sgRNAs. 2](#_Toc524954363)

[Supplementary Figure 1: Response curves of inducible systems. 3](#_Toc524954364)

[Supplementary Figure 2: Numbers of cells per ml as a function of optical density (OD_600_). 4](#_Toc524954365)

[Supplementary Figure 3: Immunoblotting and protein number estimation. 7](#_Toc524954366)

[Supplementary Figure 4: Sensitivity of dCas9*_PhlF to the addition of DAPG. 8](#_Toc524954367)

[Supplementary Figure 5: Four inducible systems that respond to small molecules. 9](#_Toc524954368)

[Supplementary Figure 6: Representative histograms corresponding to the cascades. 10](#_Toc524954369)

[Supplementary Figure 7: Evaluation of cascades at lower dCas9*_PhlF expression. 11](#_Toc524954370)

[Supplementary Figure 8: Plasmids with different numbers of sgRNAs. 12](#_Toc524954371)

[Supplementary Figure 9: −/+ sgRNA fold-change of the cognate promoter. 13](#_Toc524954372)

[Supplementary Figure 10: Toxicity of expressing multiple sgRNAs. 14](#_Toc524954373)

[Supplementary Figure 11: Sequences of 30 sgRNA and cognate promoters. 15](#_Toc524954374)

[Supplementary Figure 12: Plasmid maps for gate components. 16](#_Toc524954375)

[Supplementary Figure 13: Plasmid maps for circuit characterization. 18](#_Toc524954376)

[Supplementary Table 1: Measured gate parameters ^a^. 19](#_Toc524954377)

[Supplementary Table 2: Sequences of promoters used in Figure 1C. 20](#_Toc524954378)

[Supplementary Table 3: Sequences of genetic parts used in this study. 21](#_Toc524954379)

[Supplementary References: 29](#_Toc524954380)

**Supplementary Note: Derivation for the impact of dCas9 sharing by multiple sgRNAs.**

When multiple competing sgRNAs ($i=2\ldots n$) are expressed, we have:

$C_{TOT}=C_{F}+C_{s1}+\sum_{i=2}^{n} C_{si}$ , (1)

$\frac{ds_{1}}{dt}=\alpha_{1}-\delta_{s}s_{1}-k_{1}C_{F}s_{1}+k_{-1}C_{s1}$ , (2)

$\frac{ds_{i}}{dt}=\alpha_{i}-\delta_{s}s_{i}-k_{1}C_{F}s_{i}+k_{-1}C_{si}$ . (3)

We assume all the co-expressed competing sgRNAs have the same transcription rate *α_i_* =*α_x_* for *i* = 2…*n*.

For the formation of each sgRNA::dCas9 complex:

$\frac{dC_{s1}}{dt}={k_{1}C}_{F}s_{1}-k_{-1}C_{s1}$ , (4)

$\frac{dC_{si}}{dt}={k_{1}C}_{F}s_{i}-k_{-1}C_{si}$ . (5)

And the dynamics of free dCas9 is given by:

$\frac{dC_{F}}{dt}={-k_{1}C}_{F}s_{1}-\sum_{i=2}^{n} k_{1}C_{F}s_{i}+k_{-1}C_{s1} {+ \sum_{i=2}^{n} k_{-1}C}_{si}$ . (6)

At steady-state, Equations 1-6 reduce to

$s_{1}=\frac{\alpha_{1}}{\delta_{s}}$ and $s_{i}=\frac{\alpha_{X}}{\delta_{s}}$ , (7)

$C_{s1}=\frac{\alpha_{1}C_{TOT}}{\beta+\alpha_{1}+N\alpha_{X}}$ , (8)

where $\beta= \frac{\delta_{s}}{K_{1}}$ ,

and $N=n-1$ is the number of co-expressed competing sgRNAs.

**Supplementary Figure 1: Response curves of inducible systems.**

From left to right: pSZ_pTet, pSZ_Input, pSZ_Sensor (Supplementary Figure 12 and 13). The solid line in each figure is a fit to a Hill equation. The pTet promoter activities were used to compare the expression levels of dCas9 in Figure 1D and 1F. The average of three experiments performed on different days is shown and the error bars indicate the standard deviation.

**Supplementary Figure 2: Numbers of cells per ml as a function of optical density (OD_600_).**

These data are used to calculate protein concentrations. After growth, aliquots were diluted 2 × 10^7^-fold and plated on LB agar (Methods). The colony numbers were then counted after overnight growth at 37 °C. A linear regression curve ($y$ = 8.7 × 10^8^$x$) was fit to these data and used to calculate protein numbers per cell. The average of three experiments performed on different days is shown and the error bars indicate the standard deviation.

| Well number | Sample ^a^ | Cas9 volume (μl) | Total volume (μl) | Cas9s in well ^b^ | Band intensity |
| --- | --- | --- | --- | --- | --- |
| 1 | Cas9 standard | 0.2 | 20 | 6.02 x 10^9^ | 0.472 |
| 2 | Cas9 standard | 1.0 | 20 | 3.01 x 10^10^ | 1.545 |
| 3 | Cas9 standard | 3.0 | 20 | 9.03 x 10^10^ | 3.548 |
| 4 | Cas9 standard | 5.0 | 20 | 1.50 x 10^11^ | 4.852 |

a. Concentration of Cas9 standard is 50 nM.

b. In the well with 0.2 μl Cas9 standard added, the Cas9 number is: 50 nM × 0.2 μl × 6.02 × 10^23^ = 6.02 × 10^9^

| Well number | Sample | Lysate  volume (μl) | Total  volume (μl) | Cells  in well | Band  intensity | dCas9s  in well | dCas9s  per cell ^c^ |
| --- | --- | --- | --- | --- | --- | --- | --- |
| 5 | dCas9 | 3.00 | 20 | 4.499 x 10^7^ | 1.255 | 2.358 x 10^10^ | 524 |
| 6 | dCas9*_PhlF | 0.75 | 20 | 1.124 x 10^7^ | 3.849 | 1.041 x 10^11^ | 9260 |

c. 40 μl cell lysate was prepared from 700 μl *E. coli* culture of OD_600nm_ = 1. In the well with 3 μl cell lysate added, cell number in that well is : 8.57 × 10^8^ × 0.7 × 3 μl / 40 μl = 4.499 × 10^7^. dCas9s per cell can then be calculated, which is: 2.358 x 10^10^ / (4.499 x 10^7^) = 524.

| Well number | Sample | Cas9 volume (μl) | Total volume (μl) | Cas9s in well | Band intensity |
| --- | --- | --- | --- | --- | --- |
| 1 | Cas9 standard | 0.2 | 20 | 6.02 x 10^9^ | 0.978 |
| 2 | Cas9 standard | 1.0 | 20 | 3.01 x 10^10^ | 2.758 |
| 3 | Cas9 standard | 3.0 | 20 | 9.03 x 10^10^ | 5.456 |
| 4 | Cas9 standard | 5.0 | 20 | 1.50 x 10^11^ | 7.304 |

| Well number | Sample | Lysate  volume (μl) | Total  volume (μl) | Cells  in well | Band  intensity | dCas9s  in well | dCas9s  per cell |
| --- | --- | --- | --- | --- | --- | --- | --- |
| 5 | dCas9 | 3.00 | 20 | 4.499 x 10^7^ | 2.447 | 2.588 x 10^10^ | 575 |
| 6 | dCas9*_PhlF | 0.75 | 20 | 1.124 x 10^7^ | 6.296 | 1.176 x 10^11^ | 10459 |

| Well number | Sample | Cas9 volume (μl) | Total volume (μl) | Cas9s in well | Band intensity |
| --- | --- | --- | --- | --- | --- |
| 1 | Cas9 standard | 0.2 | 20 | 6.02 x 10^9^ | 0.238 |
| 2 | Cas9 standard | 1.0 | 20 | 3.01 x 10^10^ | 1.084 |
| 3 | Cas9 standard | 3.0 | 20 | 9.03 x 10^10^ | 2.533 |
| 4 | Cas9 standard | 5.0 | 20 | 1.50 x 10^11^ | 4.163 |

| Well number | Sample | Lysate  volume (μl) | Total  volume (μl) | Cells  in well | Band  intensity | dCas9s  in well | dCas9s  per cell |
| --- | --- | --- | --- | --- | --- | --- | --- |
| 5 | dCas9 | 3.00 | 20 | 4.499 x 10^7^ | 0.798 | 2.195 x 10^10^ | 487 |
| 6 | dCas9*_PhlF | 0.75 | 20 | 1.124 x 10^7^ | 2.849 | 1.019 x 10^11^ | 9065 |

**Supplementary Figure 3: Immunoblotting and protein number estimation.**

In each figure, green rectangles represent the band area that are used to obtain the standard curve and sample immunoblotting intensities. Blue rectangles are the area used to correct for background. Black rectangle in the first image is the area presented in Figure 1G.

**Supplementary Figure 4:** **Sensitivity of dCas9*_PhlF to the addition of DAPG.**

The fold-repression is shown in the absence (black bars) and presence (white bars) of the PhlF inducer DAPG (100 μM). The pSZ_Output and pSZ_PhlF plasmids were used for these experiments (Supplementary Figure 12). The average of three experiments performed on different days is shown and the error bars indicate the standard deviation.

**Supplementary Figure 5:** **Four inducible systems that respond to small molecules.**

White bars are the output promoter strength without inducers, and black bars are the output promoter strength when each inducer was added (measured with plasmid pSZ_Sensor, Supplementary Figure 13). The inducer concentrations used to fully induce the promoters (from left to right): 1 mM IPTG, 100 μM vanillic acid, 10 mM Choline, and 10 μM 3OC6-AHL. The average of three experiments performed on different days is shown and the error bars indicate the standard deviation.

**Supplementary Figure 6: Representative histograms corresponding to the cascades.**

Distributions are shown for the cascades in the presence (+) and absence (−) of inducer (100 μM vanillic acid). These data correspond to Figure 2D.

**Supplementary Figure 7:** **Evaluation of cascades at lower dCas9*_PhlF expression.**

The same experiments described in Figure 2D were repeated, but where dCas9*_PhlF is expressed at a lower level. The inducer concentration was 0.5 ng/ml aTc. The average of three experiments performed on different days is shown and the error bars indicate the standard deviation.

**Supplementary Figure 8:** **Plasmids with different numbers of sgRNAs.**

Verification of the sizes of constructs shown in Figure 3B. M: DNA ladder; N1-N16: Plasmids containing 1 to 16 sgRNAs (Supplementary Figure 13). These plasmids were all digested with BspHI to linearize the plasmids. Expected sizes of these linearized plasmids after digestion are (from left to right): 2300 bp, 2914 bp, 3351 bp, 3572 bp, 4023 bp, 4467 bp, 4834 bp, 5333 bp, and 5773 bp.

**Supplementary Figure 9: −/+ sgRNA fold-change of the cognate promoter.**

Each strain was transformed with a plasmid contains the cognate promoter (pSZ_Gate, Supplementary Figure 13) for each titration sgRNA. −/+ sgRNA fold-change of the promoter was then measured by co-transforming each strain with the plasmid (pSZ_Titration, Supplementary Figure 13) contains all 16 titration sgRNAs. The average of three experiments performed on different days is shown and the error bars indicate the standard deviation.

**Supplementary Figure 10: Toxicity of expressing multiple sgRNAs.**

The growth impact of co-expressing multiple sgRNAs was compared and normalized to the strain with no sgRNA expressed, following the same growth assay as in Figure 1D (Methods). No dCas9 or dCas9*_PhlF was expressed in these experiments. The average of three experiments performed on different days is shown and the error bars indicate the standard deviation.

**
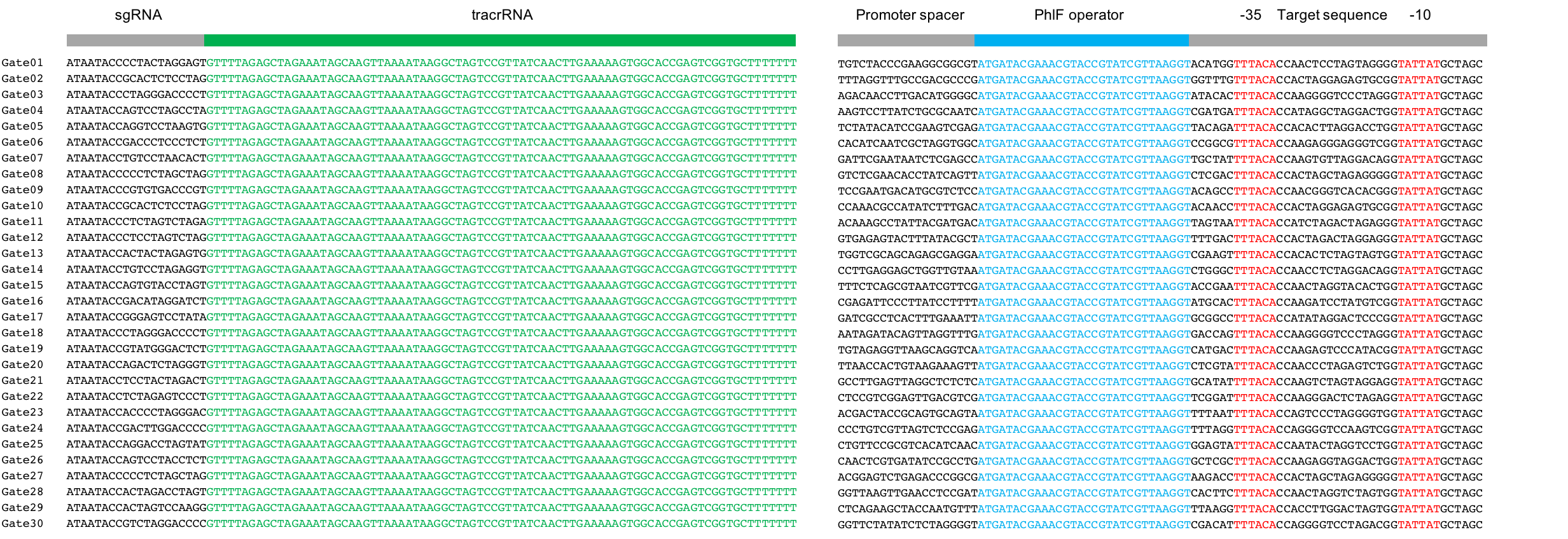
**

**Supplementary Figure 11: Sequences of 30 sgRNA and cognate promoters.**

sgRNA is the seed region that targets the cognate promoter (Target sequence) and tracrRNA is the scaffold region of sgRNA. All promoters have the same 30 bp PhlF operator sequence and additional 20 bp random generated spacer sequence (Promoter spacer) (Methods).

**Supplementary Figure 12: Plasmid maps for gate components.**

pSZ_Backgbone: Plasmid used to measure auto-fluorescence; pSZ_pTet: Plasmid for measuring pTet promoter strength, which is used to quantify dCas9 expression levels in Figure 1D and 1F; pSZ_Output: Plasmid for measuring output promoter strength; pSZ_Input: Plasmid for measuring input promoter (pTac) strength; Input promoter and output promoter strengths are combined to generate the response curves in Figure 2. pSZ_ZFP: Plasmid with fused dCas9*_ZFP complex; pSZ_PhlF: Plasmid expressing the fused dCas9*_PhlF.

**Supplementary Figure 13: Plasmid maps for circuit characterization.**

pSZ_Sensor: Four input sensor plasmid used to measure the input gates parameters; pSZ_Gate: Plasmid used to measure gate parameters; pSZ_NOT1: 1-layer NOT inverter; pSZ_NOT2: 2-layer NOT inverter; pSZ_NOT3: 3-layer NOT inverter; pSZ_NOT4: 4-layer NOT inverter; pSZ-RT1 and pSZ-RT2: Plasmids for measuring retroactivity in Figure 3A. pSZ-Titration: Plasmid for expressing sgRNA arrays.

**Supplementary Table 1: Measured gate parameters ^a^.**

| **Gate ^b^** | **Y_min_** | **Y_max_** | **K** | **n** |
| --- | --- | --- | --- | --- |
| Gate01 | 7.3 | 280 | 36 | 1.5 |
| Gate02 | 8.7 | 470 | 19 | 1.7 |
| Gate03 | 27 | 1100 | 88 | 1.4 |
| Gate04 | 5.8 | 670 | 42 | 1.3 |
| Gate05 | 6.4 | 510 | 26 | 1.5 |
| Gate06 | 3.6 | 200 | 47 | 1.5 |
| Gate07 | 2.8 | 170 | 23 | 1.6 |
| Gate08 | 9.8 | 740 | 21 | 1.6 |
| Gate09 | 10 | 710 | 25 | 1.4 |
| Gate10 | 10 | 609 | 49 | 1.5 |
| Gate11 | 5.3 | 260 | 34 | 1.6 |
| Gate12 | 23 | 500 | 42 | 1.6 |
| Gate13 | 1.4 | 230 | 80 | 1.8 |
| Gate14 | 1.9 | 420 | 85 | 1.8 |
| Gate15 | 0.9 | 150 | 59 | 1.8 |
| Gate16 | 1.2 | 340 | 82 | 1.7 |
| Gate17 | 1.4 | 320 | 27 | 1.4 |
| Gate18 | 0.9 | 450 | 103 | 1.7 |
| Gate19 | 1.5 | 590 | 77 | 1.7 |
| Gate20 | 2.5 | 670 | 68 | 1.5 |
| Gate21 | 0.2 | 130 | 63 | 1.7 |
| Gate22 | 0.4 | 160 | 56 | 1.7 |
| Gate23 | 0.3 | 370 | 72 | 1.7 |
| Gate24 | 2.1 | 450 | 89 | 1.7 |
| Gate25 | 2.3 | 410 | 73 | 1.8 |
| Gate26 | 1.2 | 500 | 78 | 1.7 |
| Gate27 | 2.0 | 250 | 64 | 1.5 |
| Gate28 | 0.9 | 570 | 56 | 1.5 |
| Gate29 | 5.8 | 670 | 104 | 1.7 |
| Gate30 | 2.2 | 110 | 63 | 1.7 |

1. Parameters are shown for a fit to Equation 1 in main text.
2. Sequences are provided in Supplementary Figure 11.

**Supplementary Table 2: Sequences of promoters used in Figure 1C.**

| **Part name** | **Type** | **DNA Sequence** |
| --- | --- | --- |
| pZFP_F | Promoter | TCCGAATGACATGCGTCTCCCGCTCCAACACCGTTGGTTGAACAGCCTTTACACCAACGGGTCACACGGGTATTATGCTAGC |
| pZFP_R | Promoter | TCCGAATGACATGCGTCTCCGGTGTTGGAGCGGTTGGTTGAACAGCCTTTACACCAACGGGTCACACGGGTATTATGCTAGC |
| pZFP_S0 | Promoter | TCCGAATGACATGCGTCTCCGGTGTTGGAGCGTTTACACCAACGGGTCACACGGGTATTATGCTAGC |
| pZFP_S2 | Promoter | TCCGAATGACATGCGTCTCCGGTGTTGGAGCGCCTTTACACCAACGGGTCACACGGGTATTATGCTAGC |
| pZFP_S4 | Promoter | TCCGAATGACATGCGTCTCCGGTGTTGGAGCGAGCCTTTACACCAACGGGTCACACGGGTATTATGCTAGC |
| pZFP_S6 | Promoter | TCCGAATGACATGCGTCTCCGGTGTTGGAGCGACAGCCTTTACACCAACGGGTCACACGGGTATTATGCTAGC |
| pZFP_S8 | Promoter | TCCGAATGACATGCGTCTCCGGTGTTGGAGCGGAACAGCCTTTACACCAACGGGTCACACGGGTATTATGCTAGC |
| pPhlF_S2 | Promoter | TCCGAATGACATGCGTCTCCATGATACGAAACGTACCGTATCGTTAAGGTCCTTTACACCAACGGGTCACACGGGTATTATGCTAGC |
| pPhlF_S4 | Promoter | TCCGAATGACATGCGTCTCCATGATACGAAACGTACCGTATCGTTAAGGTAGCCTTTACACCAACGGGTCACACGGGTATTATGCTAGC |
| pPhlF_S5 | Promoter | TCCGAATGACATGCGTCTCCATGATACGAAACGTACCGTATCGTTAAGGTCAGCCTTTACACCAACGGGTCACACGGGTATTATGCTAGC |
| pPhlF_S6 | Promoter | TCCGAATGACATGCGTCTCCATGATACGAAACGTACCGTATCGTTAAGGTACAGCCTTTACACCAACGGGTCACACGGGTATTATGCTAGC |
| pPhlF_S7 | Promoter | TCCGAATGACATGCGTCTCCATGATACGAAACGTACCGTATCGTTAAGGTAACAGCCTTTACACCAACGGGTCACACGGGTATTATGCTAGC |
| pPhlF_S15 | Promoter | TCCGAATGACATGCGTCTCCATGATACGAAACGTACCGTATCGTTAAGGTGTTGGTTGAACAGCCTTTACACCAACGGGTCACACGGGTATTATGCTAGC |
| pSrpR | Promoter | TCCGAATGACATGCGTCTCCATATACATACATGCTTGTTTGTTTGTAAACACAGCCTTTACACCAACGGGTCACACGGGTATTATGCTAGC |
| pHlyIIR | Promoter | TCCGAATGACATGCGTCTCCATATTTAAAATTCTTGTTTAAAACAGCCTTTACACCAACGGGTCACACGGGTATTATGCTAGC |
| pBM3RI | Promoter | TCCGAATGACATGCGTCTCCCGGAATGAACGTTCATTCCGACAGCCTTTACACCAACGGGTCACACGGGTATTATGCTAGC |

**Supplementary Table 3: Sequences of genetic parts used in this study.**

| **Part name** | **Type** | **DNA Sequence** |
| --- | --- | --- |
| J23101 | Promoter | TTTACAGCTAGCTCAGTCCTAGGTATTATGCTAGC |
| pCon | Promoter | TTTACACCAACTCCTAGTAGGGGTATTATGCTAGC |
| pTac | Promoter | TGTTGACAATTAATCATCGGCTCGTATAATGTGTGGAATTGTGAGCGCTCACAATT |
| pVan | Promoter | ATTGGATCCAATTGACAGCTAGCTCAGTCCTAGGTACCATTGGATCCAAT |
| pBetI | Promoter | AGCGCGGGTGAGAGGGATTCGTTACCAATAGACAATTGATTGGACGTTCAATATAATGCTAGC |
| pLuxR | Promoter | ACCTGTAGGATCGTACAGGTTTACGCAAGAAAATGGTTTGTTACAGTCGAATAAA |
| pTet | Promoter | TACTCCACCGTTGGCTTTTTTCCCTATCAGTGATAGAGATTGACATCCCTATCAGTGATAGAGATAATGAGCAC |
| N1 | sgRNA array | TTTACACCAACTCCTAGTAGGGGTATTATGCTAGCATAATACCGCACTCTCCTAGGTTTTAGAGCTAGAAATAGCAAGTTAAAATAAGGCTAGTCCGTTATCAACTTGAAAAAGTGGCACCGAGTCGGTGCTTTTTTTCTCGGTACCAAATTTTCGAAAAAAGACGCTGAAAAGCGTCTTTTTTCGTTTTGGTCC |
| N3 | sgRNA array | TTTACACCAACTCCTAGTAGGGGTATTATGCTAGCATAATACCGCACTCTCCTAGGTTTTAGAGCTAGAAATAGCAAGTTAAAATAAGGCTAGTCCGTTATCAACTTGAAAAAGTGGCACCGAGTCGGTGCTTTTTTTCTCGGTACCAAATTTTCGAAAAAAGACGCTGAAAAGCGTCTTTTTTCGTTTTGGTCCccaaacgccatatctttgacTCCGTTAACGGTCACGAGTTTTTACACCAACTCCTAGTAGGGGTATTATGCTAGCATAATACCTGTCCTAGAGGTGTTTTAGAGCTAGAAATAGCAAGTTAAAATAAGGCTAGTCCGTTATCAACTTGAAAAAGTGGCACCGAGTCGGTGCTTTTTTTAAAAAAAAAAAAGGCCTCCCAAATCGGGGGGCCTTTTTTATTGATAACAAAAccttgaggagctggttgtaaTTTACACCAACTCCTAGTAGGGGTATTATGCTAGCATAATACCAGTGTACCTAGTGTTTTAGAGCTAGAAATAGCAAGTTAAAATAAGGCTAGTCCGTTATCAACTTGAAAAAGTGGCACCGAGTCGGTGCTTTTTTTCTCGGTACCAAATTCCAGAAAAGAGACGCTTAACAGCGTCTTTTTTCGTTTTGGTCC |
| N5 | sgRNA array | TTTACACCAACTCCTAGTAGGGGTATTATGCTAGCATAATACCGCACTCTCCTAGGTTTTAGAGCTAGAAATAGCAAGTTAAAATAAGGCTAGTCCGTTATCAACTTGAAAAAGTGGCACCGAGTCGGTGCTTTTTTTCTCGGTACCAAATTTTCGAAAAAAGACGCTGAAAAGCGTCTTTTTTCGTTTTGGTCCccaaacgccatatctttgacTCCGTTAACGGTCACGAGTTTTTACACCAACTCCTAGTAGGGGTATTATGCTAGCATAATACCTGTCCTAGAGGTGTTTTAGAGCTAGAAATAGCAAGTTAAAATAAGGCTAGTCCGTTATCAACTTGAAAAAGTGGCACCGAGTCGGTGCTTTTTTTAAAAAAAAAAAAGGCCTCCCAAATCGGGGGGCCTTTTTTATTGATAACAAAAccttgaggagctggttgtaaTTTACACCAACTCCTAGTAGGGGTATTATGCTAGCATAATACCAGTGTACCTAGTGTTTTAGAGCTAGAAATAGCAAGTTAAAATAAGGCTAGTCCGTTATCAACTTGAAAAAGTGGCACCGAGTCGGTGCTTTTTTTCTCGGTACCAAATTCCAGAAAAGAGACGCTTAACAGCGTCTTTTTTCGTTTTGGTCCtttctcagcgtaatcgttcgCGAAATCGAAGGTGAAGGTGTTTACACCAACTCCTAGTAGGGGTATTATGCTAGCATAATACCGACATAGGATCTGTTTTAGAGCTAGAAATAGCAAGTTAAAATAAGGCTAGTCCGTTATCAACTTGAAAAAGTGGCACCGAGTCGGTGCTTTTTTTCCAATTATTGAAGGCCGCTAACGCGGCCTTTTTTTGTTTCTGGTCTCCCcgagattcccttatccttttTTTACACCAACTCCTAGTAGGGGTATTATGCTAGCATAATACCGGGAGTCCTATAGTTTTAGAGCTAGAAATAGCAAGTTAAAATAAGGCTAGTCCGTTATCAACTTGAAAAAGTGGCACCGAGTCGGTGCTTTTTTTCCAATTATTGAAGGCCTCCCAAATCGGGGGGCCTTTTTTATTGATAACAAAA |
| N6 | sgRNA array | TTTACACCAACTCCTAGTAGGGGTATTATGCTAGCATAATACCTGTCCTAGAGGTGTTTTAGAGCTAGAAATAGCAAGTTAAAATAAGGCTAGTCCGTTATCAACTTGAAAAAGTGGCACCGAGTCGGTGCTTTTTTTAAAAAAAAAAAAGGCCTCCCAAATCGGGGGGCCTTTTTTATTGATAACAAAAccttgaggagctggttgtaaTTTACACCAACTCCTAGTAGGGGTATTATGCTAGCATAATACCAGTGTACCTAGTGTTTTAGAGCTAGAAATAGCAAGTTAAAATAAGGCTAGTCCGTTATCAACTTGAAAAAGTGGCACCGAGTCGGTGCTTTTTTTCTCGGTACCAAATTCCAGAAAAGAGACGCTTAACAGCGTCTTTTTTCGTTTTGGTCCtttctcagcgtaatcgttcgCGAAATCGAAGGTGAAGGTGTTTACACCAACTCCTAGTAGGGGTATTATGCTAGCATAATACCGACATAGGATCTGTTTTAGAGCTAGAAATAGCAAGTTAAAATAAGGCTAGTCCGTTATCAACTTGAAAAAGTGGCACCGAGTCGGTGCTTTTTTTCCAATTATTGAAGGCCGCTAACGCGGCCTTTTTTTGTTTCTGGTCTCCCcgagattcccttatccttttTTTACACCAACTCCTAGTAGGGGTATTATGCTAGCATAATACCGGGAGTCCTATAGTTTTAGAGCTAGAAATAGCAAGTTAAAATAAGGCTAGTCCGTTATCAACTTGAAAAAGTGGCACCGAGTCGGTGCTTTTTTTCCAATTATTGAAGGCCTCCCAAATCGGGGGGCCTTTTTTATTGATAACAAAAgatcgcctcactttgaaattTATCAAAGAGTTCATGCGTTTTTACACCAACTCCTAGTAGGGGTATTATGCTAGCATAATACCCTAGGGACCCCTGTTTTAGAGCTAGAAATAGCAAGTTAAAATAAGGCTAGTCCGTTATCAACTTGAAAAAGTGGCACCGAGTCGGTGCTTTTTTTCTCGGTACCAAAAAAAAAAAAAAAGACGCTGAAAAGCGTCTTTTTTCGTTTTGGTCCaatagatacagttaggtttgTTTACACCAACTCCTAGTAGGGGTATTATGCTAGCATAATACCCCCTCTAGCTAGGTTTTAGAGCTAGAAATAGCAAGTTAAAATAAGGCTAGTCCGTTATCAACTTGAAAAAGTGGCACCGAGTCGGTGCTTTTTTTCTCGGTACCAAAAAAAAAAAAAAAGACGCTGAAAAGCGTCTTTTTTTTTTTTGGTCC |
| N8 | sgRNA array | TTTACACCAACTCCTAGTAGGGGTATTATGCTAGCATAATACCTGTCCTAGAGGTGTTTTAGAGCTAGAAATAGCAAGTTAAAATAAGGCTAGTCCGTTATCAACTTGAAAAAGTGGCACCGAGTCGGTGCTTTTTTTAAAAAAAAAAAAGGCCTCCCAAATCGGGGGGCCTTTTTTATTGATAACAAAAccttgaggagctggttgtaaTTTACACCAACTCCTAGTAGGGGTATTATGCTAGCATAATACCAGTGTACCTAGTGTTTTAGAGCTAGAAATAGCAAGTTAAAATAAGGCTAGTCCGTTATCAACTTGAAAAAGTGGCACCGAGTCGGTGCTTTTTTTCTCGGTACCAAATTCCAGAAAAGAGACGCTTAACAGCGTCTTTTTTCGTTTTGGTCCtttctcagcgtaatcgttcgCGAAATCGAAGGTGAAGGTGTTTACACCAACTCCTAGTAGGGGTATTATGCTAGCATAATACCGACATAGGATCTGTTTTAGAGCTAGAAATAGCAAGTTAAAATAAGGCTAGTCCGTTATCAACTTGAAAAAGTGGCACCGAGTCGGTGCTTTTTTTCCAATTATTGAAGGCCGCTAACGCGGCCTTTTTTTGTTTCTGGTCTCCCcgagattcccttatccttttTTTACACCAACTCCTAGTAGGGGTATTATGCTAGCATAATACCGGGAGTCCTATAGTTTTAGAGCTAGAAATAGCAAGTTAAAATAAGGCTAGTCCGTTATCAACTTGAAAAAGTGGCACCGAGTCGGTGCTTTTTTTCCAATTATTGAAGGCCTCCCAAATCGGGGGGCCTTTTTTATTGATAACAAAAgatcgcctcactttgaaattTATCAAAGAGTTCATGCGTTTTTACACCAACTCCTAGTAGGGGTATTATGCTAGCATAATACCCTAGGGACCCCTGTTTTAGAGCTAGAAATAGCAAGTTAAAATAAGGCTAGTCCGTTATCAACTTGAAAAAGTGGCACCGAGTCGGTGCTTTTTTTCTCGGTACCAAAAAAAAAAAAAAAGACGCTGAAAAGCGTCTTTTTTCGTTTTGGTCCaatagatacagttaggtttgTTTACACCAACTCCTAGTAGGGGTATTATGCTAGCATAATACCCCCTCTAGCTAGGTTTTAGAGCTAGAAATAGCAAGTTAAAATAAGGCTAGTCCGTTATCAACTTGAAAAAGTGGCACCGAGTCGGTGCTTTTTTTCTCGGTACCAAAAAAAAAAAAAAAGACGCTGAAAAGCGTCTTTTTTTTTTTTGGTCCacggagtctgagacTcggcgAAGGTCGTCCGTACGAAGGTTTTACACCAACTCCTAGTAGGGGTATTATGCTAGCATAATACCGTATGGGACTCTGTTTTAGAGCTAGAAATAGCAAGTTAAAATAAGGCTAGTCCGTTATCAACTTGAAAAAGTGGCACCGAGTCGGTGCTTTTTTTCTCGGTACCAAACCAATTATTGAAGACGCTGAAAAGCGTCTTTTTTCGTTTTGGTCCtgtagaggttaagcaggtcaTTTACACCAACTCCTAGTAGGGGTATTATGCTAGCATAATACCAGACTCTAGGGTGTTTTAGAGCTAGAAATAGCAAGTTAAAATAAGGCTAGTCCGTTATCAACTTGAAAAAGTGGCACCGAGTCGGTGCTTTTTTTCTCGGTACCAAATTCCAGAAAAGAGACGCTTTTAGAGCGTCTTTTTTCGTTTTGGTCC |
| N10 | sgRNA array | TTTACACCAACTCCTAGTAGGGGTATTATGCTAGCATAATACCTGTCCTAGAGGTGTTTTAGAGCTAGAAATAGCAAGTTAAAATAAGGCTAGTCCGTTATCAACTTGAAAAAGTGGCACCGAGTCGGTGCTTTTTTTAAAAAAAAAAAAGGCCTCCCAAATCGGGGGGCCTTTTTTATTGATAACAAAAccttgaggagctggttgtaaTTTACACCAACTCCTAGTAGGGGTATTATGCTAGCATAATACCAGTGTACCTAGTGTTTTAGAGCTAGAAATAGCAAGTTAAAATAAGGCTAGTCCGTTATCAACTTGAAAAAGTGGCACCGAGTCGGTGCTTTTTTTCTCGGTACCAAATTCCAGAAAAGAGACGCTTAACAGCGTCTTTTTTCGTTTTGGTCCtttctcagcgtaatcgttcgCGAAATCGAAGGTGAAGGTGTTTACACCAACTCCTAGTAGGGGTATTATGCTAGCATAATACCGACATAGGATCTGTTTTAGAGCTAGAAATAGCAAGTTAAAATAAGGCTAGTCCGTTATCAACTTGAAAAAGTGGCACCGAGTCGGTGCTTTTTTTCCAATTATTGAAGGCCGCTAACGCGGCCTTTTTTTGTTTCTGGTCTCCCcgagattcccttatccttttTTTACACCAACTCCTAGTAGGGGTATTATGCTAGCATAATACCGGGAGTCCTATAGTTTTAGAGCTAGAAATAGCAAGTTAAAATAAGGCTAGTCCGTTATCAACTTGAAAAAGTGGCACCGAGTCGGTGCTTTTTTTCCAATTATTGAAGGCCTCCCAAATCGGGGGGCCTTTTTTATTGATAACAAAAgatcgcctcactttgaaattTATCAAAGAGTTCATGCGTTTTTACACCAACTCCTAGTAGGGGTATTATGCTAGCATAATACCCTAGGGACCCCTGTTTTAGAGCTAGAAATAGCAAGTTAAAATAAGGCTAGTCCGTTATCAACTTGAAAAAGTGGCACCGAGTCGGTGCTTTTTTTCTCGGTACCAAAAAAAAAAAAAAAGACGCTGAAAAGCGTCTTTTTTCGTTTTGGTCCaatagatacagttaggtttgTTTACACCAACTCCTAGTAGGGGTATTATGCTAGCATAATACCCCCTCTAGCTAGGTTTTAGAGCTAGAAATAGCAAGTTAAAATAAGGCTAGTCCGTTATCAACTTGAAAAAGTGGCACCGAGTCGGTGCTTTTTTTCTCGGTACCAAAAAAAAAAAAAAAGACGCTGAAAAGCGTCTTTTTTTTTTTTGGTCCacggagtctgagacTcggcgAAGGTCGTCCGTACGAAGGTTTTACACCAACTCCTAGTAGGGGTATTATGCTAGCATAATACCGTATGGGACTCTGTTTTAGAGCTAGAAATAGCAAGTTAAAATAAGGCTAGTCCGTTATCAACTTGAAAAAGTGGCACCGAGTCGGTGCTTTTTTTCTCGGTACCAAACCAATTATTGAAGACGCTGAAAAGCGTCTTTTTTCGTTTTGGTCCtgtagaggttaagcaggtcaTTTACACCAACTCCTAGTAGGGGTATTATGCTAGCATAATACCAGACTCTAGGGTGTTTTAGAGCTAGAAATAGCAAGTTAAAATAAGGCTAGTCCGTTATCAACTTGAAAAAGTGGCACCGAGTCGGTGCTTTTTTTCTCGGTACCAAATTCCAGAAAAGAGACGCTTTTAGAGCGTCTTTTTTCGTTTTGGTCCttaaccactgtaagaaagttACCCAGACCGCTAAACTGAATTTACACCAACTCCTAGTAGGGGTATTATGCTAGCATAATACCTCCTACTAGACTGTTTTAGAGCTAGAAATAGCAAGTTAAAATAAGGCTAGTCCGTTATCAACTTGAAAAAGTGGCACCGAGTCGGTGCTTTTTTTCTCGGTACCAAATTCCAGAAAAGAGACGCTGAAAAGCGTCTTTTTTTTTTTTGGTCCgccttgagttaggctctctcTTTACACCAACTCCTAGTAGGGGTATTATGCTAGCATAATACCTCTAGAGTCCCTGTTTTAGAGCTAGAAATAGCAAGTTAAAATAAGGCTAGTCCGTTATCAACTTGAAAAAGTGGCACCGAGTCGGTGCTTTTTTTGACGAACAATAAGGCCTCCCTAACGGGGGGCCTTTTTTATTGATAACAAAA |
| N12 | sgRNA array | TTTACACCAACTCCTAGTAGGGGTATTATGCTAGCATAATACCTGTCCTAGAGGTGTTTTAGAGCTAGAAATAGCAAGTTAAAATAAGGCTAGTCCGTTATCAACTTGAAAAAGTGGCACCGAGTCGGTGCTTTTTTTAAAAAAAAAAAAGGCCTCCCAAATCGGGGGGCCTTTTTTATTGATAACAAAAccttgaggagctggttgtaaTTTACACCAACTCCTAGTAGGGGTATTATGCTAGCATAATACCAGTGTACCTAGTGTTTTAGAGCTAGAAATAGCAAGTTAAAATAAGGCTAGTCCGTTATCAACTTGAAAAAGTGGCACCGAGTCGGTGCTTTTTTTCTCGGTACCAAATTCCAGAAAAGAGACGGTCGTCCGTACGAAGGTTTTACACCAACTCCTAGTAGGGGTATTATGCTAGCATAATACCGTATGGGACTCTGTTTTAGAGCTAGAAATAGCAAGTTAAAATAAGGCTAGTCCGTTATCAACTTGAAAAAGTGGCACCGAGTCGGTGCTTTTTTTCTCGGTACCAAACCAATTATTGAAGACGCTGAAAAGCGTCTTTTTTCGTTTTGGTCCtgtagaggttaagcaggtcaTTTACACCAACTCCTAGTAGGGGTATTATGCTAGCATAATACCAGACTCTAGGGTGTTTTAGAGCTAGAAATAGCAAGTTAAAATAAGGCTAGTCCGTTATCAACTTGAAAAAGTGGCACCGAGTCGGTGCTTTTTTTCTCGGTACCAAATTCCAGAAAAGAGACGCTTTTAGAGCGTCTTTTTTCGTTTTGGTCCttaaccactgtaagaaagttACCCAGACCGCTAAACTGAATTTACACCAACTCCTAGTAGGGGTATTATGCTAGCATAATACCTCCTACTAGACTGTTTTAGAGCTAGAAATAGCAAGTTAAAATAAGGCTAGTCCGTTATCAACTTGAAAAAGTGGCACCGAGTCGGTGCTTTTTTTCTCGGTACCAAATTCCAGAAAAGAGACGCTGAAAAGCGTCTTTTTTTTTTTTGGTCCgccttgagttaggctctctcTTTACACCAACTCCTAGTAGGGGTATTATGCTAGCATAATACCTCTAGAGTCCCTGTTTTAGAGCTAGAAATAGCAAGTTAAAATAAGGCTAGTCCGTTATCAACTTGAAAAAGTGGCACCGAGTCGGTGCTTTTTTTGACGAACAATAAGGCCTCCCTAACGGGGGGCCTTTTTTATTGATAACAAAActccgtcggagttgacgtcgTGCCGTTCGCTTGGGACATCTTTACACCAACTCCTAGTAGGGGTATTATGCTAGCATAATACCAGGACCTAGTATGTTTTAGAGCTAGAAATAGCAAGTTAAAATAAGGCTAGTCCGTTATCAACTTGAAAAAGTGGCACCGAGTCGGTGCTTTTTTTGACGAACAATAAGGCCTCCCGAAAGGGGGGCCTTTTTTATTGATAACAAAActgttccgcgtcacatcaacTTTACACCAACTCCTAGTAGGGGTATTATGCTAGCATAATACCAGTCCTACCTCTGTTTTAGAGCTAGAAATAGCAAGTTAAAATAAGGCTAGTCCGTTATCAACTTGAAAAAGTGGCACCGAGTCGGTGCTTTTTTTTCTAACTAAAAACACCCTAACGGGTGTTTTTTTGTTTCTGGTCTgCCcaactcgtgatatccgcctgAGTTACCAAAGGTGGTCCGCTTTACACCAACTCCTAGTAGGGGTATTATGCTAGCATAATACCACCCCTAGGGACGTTTTAGAGCTAGAAATAGCAAGTTAAAATAAGGCTAGTCCGTTATCAACTTGAAAAAGTGGCACCGAGTCGGTGCTTTTTTTCCAATTATTGAACACCCTTCGGGGTGTTTTTTTGTTTCTGGTCTCCCacgactaccgcagtgcagtaTTTACACCAACTCCTAGTAGGGGTATTATGCTAGCATAATACCGACTTGGACCCCGTTTTAGAGCTAGAAATAGCAAGTTAAAATAAGGCTAGTCCGTTATCAACTTGAAAAAGTGGCACCGAGTCGGTGCTTTTTTTCCAATTATTGAAGACGCTTAACAGCGTCTTTTTTTGTTTCTGGTCTCCCTcctgtcgttagtctccgagTCAAAGTTCGTATGGAAGGTTTTACACCAACTCCTAGTAGGGGTATTATGCTAGCATAATACCCTCCTAGTCTAGGTTTTAGAGCTAGAAATAGCAAGTTAAAATAAGGCTAGTCCGTTATCAACTTGAAAAAGTGGCACCGAGTCGGTGCTTTTTTTTTTTCGAAAAAACACCCTAACGGGTGTTTTTTTGTTTCTGGTCTCCCgtgagagtactttatacgctTTTACACCAACTCCTAGTAGGGGTATTATGCTAGCATAATACCACTACTAGAGTGGTTTTAGAGCTAGAAATAGCAAGTTAAAATAAGGCTAGTCCGTTATCAACTTGAAAAAGTGGCACCGAGTCGGTGCTTTTTTTCTCGGTACCAAATCTAACTAAAAAGACGCTGAAAAGCGTCTTTTTTCGTTTTGGTCC |
| N14 | sgRNA array | TTTACACCAACTCCTAGTAGGGGTATTATGCTAGCATAATACCTGTCCTAGAGGTGTTTTAGAGCTAGAAATAGCAAGTTAAAATAAGGCTAGTCCGTTATCAACTTGAAAAAGTGGCACCGAGTCGGTGCTTTTTTTAAAAAAAAAAAAGGCCTCCCAAATCGGGGGGCCTTTTTTATTGATAACAAAAccttgaggagctggttgtaaTTTACACCAACTCCTAGTAGGGGTATTATGCTAGCATAATACCAGTGTACCTAGTGTTTTAGAGCTAGAAATAGCAAGTTAAAATAAGGCTAGTCCGTTATCAACTTGAAAAAGTGGCACCGAGTCGGTGCTTTTTTTCTCGGTACCAAATTCCAGAAAAGAGACGCTTAACAGCGTCTTTTTTCGTTTTGGTCCtttctcagcgtaatcgttcgCGAAATCGAAGGTGAAGGTGTTTACACCAACTCCTAGTAGGGGTATTATGCTAGCATAATACCGACATAGGATCTGTTTTAGAGCTAGAAATAGCAAGTTAAAATAAGGCTAGTCCGTTATCAACTTGAAAAAGTGGCACCGAGTCGGTGCTTTTTTTCCAATTATTGAAGGCCGCTAACGCGGCCTTTTTTTGTTTCTGGTCTCCCcgagattcccttatccttttTTTACACCAACTCCTAGTAGGGGTATTATGCTAGCATAATACCGGGAGTCCTATAGTTTTAGAGCTAGAAATAGCAAGTTAAAATAAGGCTAGTCCGTTATCAACTTGAAAAAGTGGCACCGAGTCGGTGCTTTTTTTCCAATTATTGAAGGCCTCCCAAATCGGGGGGCCTTTTTTATTGATAACAAAAgatcgcctcactttgaaattTATCAAAGAGTTCATGCGTTTTTACACCAACTCCTAGTAGGGGTATTATGCTAGCATAATACCCTAGGGACCCCTGTTTTAGAGCTAGAAATAGCAAGTTAAAATAAGGCTAGTCCGTTATCAACTTGAAAAAGTGGCACCGAGTCGGTGCTTTTTTTCTCGGTACCAAAAAAAAAAAAAAAGACGCTGAAAAGCGTCTTTTTTCGTTTTGGTCCaatagatacagttaggtttgTTTACACCAACTCCTAGTAGGGGTATTATGCTAGCATAATACCCCCTCTAGCTAGGTTTTAGAGCTAGAAATAGCAAGTTAAAATAAGGCTAGTCCGTTATCAACTTGAAAAAGTGGCACCGAGTCGGTGCTTTTTTTCTCGGTACCAAAAAAAAAAAAAAAGACGCTGAAAAGCGTCTTTTTTTTTTTTGGTCCacggagtctgagacTcggcgAAGGTCGTCCGTACGAAGGTTTTACACCAACTCCTAGTAGGGGTATTATGCTAGCATAATACCGTATGGGACTCTGTTTTAGAGCTAGAAATAGCAAGTTAAAATAAGGCTAGTCCGTTATCAACTTGAAAAAGTGGCACCGAGTCGGTGCTTTTTTTCTCGGTACCAAACCAATTATTGAAGACGCTGAAAAGCGTCTTTTTTCGTTTTGGTCCtgtagaggttaagcaggtcaTTTACACCAACTCCTAGTAGGGGTATTATGCTAGCATAATACCAGACTCTAGGGTGTTTTAGAGCTAGAAATAGCAAGTTAAAATAAGGCTAGTCCGTTATCAACTTGAAAAAGTGGCACCGAGTCGGTGCTTTTTTTCTCGGTACCAAATTCCAGAAAAGAGACGCTTTTAGAGCGTCTTTTTTCGTTTTGGTCCttaaccactgtaagaaagttACCCAGACCGCTAAACTGAATTTACACCAACTCCTAGTAGGGGTATTATGCTAGCATAATACCTCCTACTAGACTGTTTTAGAGCTAGAAATAGCAAGTTAAAATAAGGCTAGTCCGTTATCAACTTGAAAAAGTGGCACCGAGTCGGTGCTTTTTTTCTCGGTACCAAATTCCAGAAAAGAGACGCTGAAAAGCGTCTTTTTTTTTTTTGGTCCgccttgagttaggctctctcTTTACACCAACTCCTAGTAGGGGTATTATGCTAGCATAATACCTCTAGAGTCCCTGTTTTAGAGCTAGAAATAGCAAGTTAAAATAAGGCTAGTCCGTTATCAACTTGAAAAAGTGGCACCGAGTCGGTGCTTTTTTTGACGAACAATAAGGCCTCCCTAACGGGGGGCCTTTTTTATTGATAACAAAActccgtcggagttgacgtcgTGCCGTTCGCTTGGGACATCTTTACACCAACTCCTAGTAGGGGTATTATGCTAGCATAATACCAGGACCTAGTATGTTTTAGAGCTAGAAATAGCAAGTTAAAATAAGGCTAGTCCGTTATCAACTTGAAAAAGTGGCACCGAGTCGGTGCTTTTTTTGACGAACAATAAGGCCTCCCGAAAGGGGGGCCTTTTTTATTGATAACAAAActgttccgcgtcacatcaacTTTACACCAACTCCTAGTAGGGGTATTATGCTAGCATAATACCAGTCCTACCTCTGTTTTAGAGCTAGAAATAGCAAGTTAAAATAAGGCTAGTCCGTTATCAACTTGAAAAAGTGGCACCGAGTCGGTGCTTTTTTTTCTAACTAAAAACACCCTAACGGGTGTTTTTTTGTTTCTGGTCTgCCcaactcgtgatatccgcctgAGTTACCAAAGGTGGTCCGCTTTACACCAACTCCTAGTAGGGGTATTATGCTAGCATAATACCACCCCTAGGGACGTTTTAGAGCTAGAAATAGCAAGTTAAAATAAGGCTAGTCCGTTATCAACTTGAAAAAGTGGCACCGAGTCGGTGCTTTTTTTCCAATTATTGAACACCCTTCGGGGTGTTTTTTTGTTTCTGGTCTCCCacgactaccgcagtgcagtaTTTACACCAACTCCTAGTAGGGGTATTATGCTAGCATAATACCGACTTGGACCCCGTTTTAGAGCTAGAAATAGCAAGTTAAAATAAGGCTAGTCCGTTATCAACTTGAAAAAGTGGCACCGAGTCGGTGCTTTTTTTCCAATTATTGAAGACGCTTAACAGCGTCTTTTTTTGTTTCTGGTCTCCC |
| N16 | sgRNA array | TTTACACCAACTCCTAGTAGGGGTATTATGCTAGCATAATACCTGTCCTAGAGGTGTTTTAGAGCTAGAAATAGCAAGTTAAAATAAGGCTAGTCCGTTATCAACTTGAAAAAGTGGCACCGAGTCGGTGCTTTTTTTAAAAAAAAAAAAGGCCTCCCAAATCGGGGGGCCTTTTTTATTGATAACAAAAccttgaggagctggttgtaaTTTACACCAACTCCTAGTAGGGGTATTATGCTAGCATAATACCAGTGTACCTAGTGTTTTAGAGCTAGAAATAGCAAGTTAAAATAAGGCTAGTCCGTTATCAACTTGAAAAAGTGGCACCGAGTCGGTGCTTTTTTTCTCGGTACCAAATTCCAGAAAAGAGACGCTTAACAGCGTCTTTTTTCGTTTTGGTCCtttctcagcgtaatcgttcgCGAAATCGAAGGTGAAGGTGTTTACACCAACTCCTAGTAGGGGTATTATGCTAGCATAATACCGACATAGGATCTGTTTTAGAGCTAGAAATAGCAAGTTAAAATAAGGCTAGTCCGTTATCAACTTGAAAAAGTGGCACCGAGTCGGTGCTTTTTTTCCAATTATTGAAGGCCGCTAACGCGGCCTTTTTTTGTTTCTGGTCTCCCcgagattcccttatccttttTTTACACCAACTCCTAGTAGGGGTATTATGCTAGCATAATACCGGGAGTCCTATAGTTTTAGAGCTAGAAATAGCAAGTTAAAATAAGGCTAGTCCGTTATCAACTTGAAAAAGTGGCACCGAGTCGGTGCTTTTTTTCCAATTATTGAAGGCCTCCCAAATCGGGGGGCCTTTTTTATTGATAACAAAAgatcgcctcactttgaaattTATCAAAGAGTTCATGCGTTTTTACACCAACTCCTAGTAGGGGTATTATGCTAGCATAATACCCTAGGGACCCCTGTTTTAGAGCTAGAAATAGCAAGTTAAAATAAGGCTAGTCCGTTATCAACTTGAAAAAGTGGCACCGAGTCGGTGCTTTTTTTCTCGGTACCAAAAAAAAAAAAAAAGACGCTGAAAAGCGTCTTTTTTCGTTTTGGTCCaatagatacagttaggtttgTTTACACCAACTCCTAGTAGGGGTATTATGCTAGCATAATACCCCCTCTAGCTAGGTTTTAGAGCTAGAAATAGCAAGTTAAAATAAGGCTAGTCCGTTATCAACTTGAAAAAGTGGCACCGAGTCGGTGCTTTTTTTCTCGGTACCAAAAAAAAAAAAAAAGACGCTGAAAAGCGTCTTTTTTTTTTTTGGTCCacggagtctgagacTcggcgAAGGTCGTCCGTACGAAGGTTTTACACCAACTCCTAGTAGGGGTATTATGCTAGCATAATACCGTATGGGACTCTGTTTTAGAGCTAGAAATAGCAAGTTAAAATAAGGCTAGTCCGTTATCAACTTGAAAAAGTGGCACCGAGTCGGTGCTTTTTTTCTCGGTACCAAACCAATTATTGAAGACGCTGAAAAGCGTCTTTTTTCGTTTTGGTCCtgtagaggttaagcaggtcaTTTACACCAACTCCTAGTAGGGGTATTATGCTAGCATAATACCAGACTCTAGGGTGTTTTAGAGCTAGAAATAGCAAGTTAAAATAAGGCTAGTCCGTTATCAACTTGAAAAAGTGGCACCGAGTCGGTGCTTTTTTTCTCGGTACCAAATTCCAGAAAAGAGACGCTTTTAGAGCGTCTTTTTTCGTTTTGGTCCttaaccactgtaagaaagttACCCAGACCGCTAAACTGAATTTACACCAACTCCTAGTAGGGGTATTATGCTAGCATAATACCTCCTACTAGACTGTTTTAGAGCTAGAAATAGCAAGTTAAAATAAGGCTAGTCCGTTATCAACTTGAAAAAGTGGCACCGAGTCGGTGCTTTTTTTCTCGGTACCAAATTCCAGAAAAGAGACGCTGAAAAGCGTCTTTTTTTTTTTTGGTCCgccttgagttaggctctctcTTTACACCAACTCCTAGTAGGGGTATTATGCTAGCATAATACCTCTAGAGTCCCTGTTTTAGAGCTAGAAATAGCAAGTTAAAATAAGGCTAGTCCGTTATCAACTTGAAAAAGTGGCACCGAGTCGGTGCTTTTTTTGACGAACAATAAGGCCTCCCTAACGGGGGGCCTTTTTTATTGATAACAAAActccgtcggagttgacgtcgTGCCGTTCGCTTGGGACATCTTTACACCAACTCCTAGTAGGGGTATTATGCTAGCATAATACCAGGACCTAGTATGTTTTAGAGCTAGAAATAGCAAGTTAAAATAAGGCTAGTCCGTTATCAACTTGAAAAAGTGGCACCGAGTCGGTGCTTTTTTTGACGAACAATAAGGCCTCCCGAAAGGGGGGCCTTTTTTATTGATAACAAAActgttccgcgtcacatcaacTTTACACCAACTCCTAGTAGGGGTATTATGCTAGCATAATACCAGTCCTACCTCTGTTTTAGAGCTAGAAATAGCAAGTTAAAATAAGGCTAGTCCGTTATCAACTTGAAAAAGTGGCACCGAGTCGGTGCTTTTTTTTCTAACTAAAAACACCCTAACGGGTGTTTTTTTGTTTCTGGTCTgCCcaactcgtgatatccgcctgAGTTACCAAAGGTGGTCCGCTTTACACCAACTCCTAGTAGGGGTATTATGCTAGCATAATACCACCCCTAGGGACGTTTTAGAGCTAGAAATAGCAAGTTAAAATAAGGCTAGTCCGTTATCAACTTGAAAAAGTGGCACCGAGTCGGTGCTTTTTTTCCAATTATTGAACACCCTTCGGGGTGTTTTTTTGTTTCTGGTCTCCCacgactaccgcagtgcagtaTTTACACCAACTCCTAGTAGGGGTATTATGCTAGCATAATACCGACTTGGACCCCGTTTTAGAGCTAGAAATAGCAAGTTAAAATAAGGCTAGTCCGTTATCAACTTGAAAAAGTGGCACCGAGTCGGTGCTTTTTTTCCAATTATTGAAGACGCTTAACAGCGTCTTTTTTTGTTTCTGGTCTCCCTcctgtcgttagtctccgagTCAAAGTTCGTATGGAAGGTTTTACACCAACTCCTAGTAGGGGTATTATGCTAGCATAATACCCTCCTAGTCTAGGTTTTAGAGCTAGAAATAGCAAGTTAAAATAAGGCTAGTCCGTTATCAACTTGAAAAAGTGGCACCGAGTCGGTGCTTTTTTTTTTTCGAAAAAACACCCTAACGGGTGTTTTTTTGTTTCTGGTCTCCCgtgagagtactttatacgctTTTACACCAACTCCTAGTAGGGGTATTATGCTAGCATAATACCACTACTAGAGTGGTTTTAGAGCTAGAAATAGCAAGTTAAAATAAGGCTAGTCCGTTATCAACTTGAAAAAGTGGCACCGAGTCGGTGCTTTTTTTCTCGGTACCAAATCTAACTAAAAAGACGCTGAAAAGCGTCTTTTTTCGTTTTGGTCC |
| RiboJ | insulator | AGCTGTCACCGGATGTGCTTTCCGGTCTGATGAGTCCGTGAGGACGAAACAGCCTCTACAAATAATTTTGTTTAA |
| *dCas9* | gene | ATGGATAAGAAATACTCAATAGGCTTAGCTATCGGCACAAATAGCGTCGGATGGGCGGTGATCACTGATGAATATAAGGTTCCGTCTAAAAAGTTCAAGGTTCTGGGAAATACAGACCGCCACAGTATCAAAAAAAATCTTATAGGGGCTCTTTTATTTGACAGTGGAGAGACAGCGGAAGCGACTCGTCTCAAACGGACAGCTCGTAGAAGGTATACACGTCGGAAGAATCGTATTTGTTATCTACAGGAGATTTTTTCAAATGAGATGGCGAAAGTAGATGATAGTTTCTTTCATCGACTTGAAGAGTCTTTTTTGGTGGAAGAAGACAAGAAGCATGAACGTCATCCTATTTTTGGAAATATAGTAGATGAAGTTGCTTATCATGAGAAATATCCAACTATCTATCATCTGCGAAAAAAATTGGTAGATTCTACTGATAAAGCGGATTTGCGCTTAATCTATTTGGCCTTAGCGCATATGATTAAGTTTCGTGGTCATTTTTTGATTGAGGGAGATTTAAATCCTGATAATAGTGATGTGGACAAACTATTTATCCAGTTGGTACAAACCTACAATCAATTATTTGAAGAAAACCCTATTAACGCAAGTGGAGTAGATGCTAAAGCGATTCTTTCTGCACGATTGAGTAAATCAAGACGATTAGAAAATCTCATTGCTCAGCTCCCCGGTGAGAAGAAAAATGGCTTATTTGGGAATCTCATTGCTTTGTCATTGGGTTTGACCCCTAATTTTAAATCAAATTTTGATTTGGCAGAAGATGCTAAATTACAGCTTTCAAAAGATACTTACGATGATGATTTAGATAATTTATTGGCGCAAATTGGAGATCAATATGCTGATTTGTTTTTGGCAGCTAAGAATTTATCAGATGCTATTTTACTTTCAGATATCCTAAGAGTAAATACTGAAATAACTAAGGCTCCCCTATCAGCTTCAATGATTAAACGCTACGATGAACATCATCAAGACTTGACTCTTTTAAAAGCTTTAGTTCGACAACAACTTCCAGAAAAGTATAAAGAAATCTTTTTTGATCAATCAAAAAACGGATATGCAGGTTATATTGATGGGGGAGCTAGCCAAGAAGAATTTTATAAATTTATCAAACCAATTTTAGAAAAAATGGATGGTACTGAGGAATTATTGGTGAAACTAAATCGTGAAGATTTGCTGCGCAAGCAACGGACCTTTGACAACGGCTCTATTCCCCATCAAATTCACTTGGGTGAGCTGCATGCTATTTTGAGAAGACAAGAAGACTTTTATCCATTTTTAAAAGACAATCGTGAGAAGATTGAAAAAATCTTGACTTTTCGAATTCCTTATTATGTTGGTCCATTGGCGCGTGGCAATAGTCGTTTTGCATGGATGACTCGGAAGTCTGAAGAAACAATTACCCCATGGAATTTTGAAGAAGTTGTCGATAAAGGTGCTTCAGCTCAATCATTTATTGAACGCATGACAAACTTTGATAAAAATCTTCCAAATGAAAAAGTACTACCAAAACATAGTTTGCTTTATGAGTATTTTACGGTTTATAACGAATTGACAAAGGTCAAATATGTTACTGAAGGAATGCGAAAACCAGCATTTCTTTCAGGTGAACAGAAGAAAGCCATTGTTGATTTACTCTTCAAAACAAATCGAAAAGTAACCGTTAAGCAATTAAAAGAAGATTATTTCAAAAAAATAGAATGTTTTGATAGTGTTGAAATTTCAGGAGTTGAAGATAGATTTAATGCTTCATTAGGTACCTACCATGATTTGCTAAAAATTATTAAAGATAAAGATTTTTTGGATAATGAAGAAAATGAAGATATCTTAGAGGATATTGTTTTAACATTGACCTTATTTGAAGATAGGGAGATGATTGAGGAAAGACTTAAAACATATGCTCACCTCTTTGATGATAAGGTGATGAAACAGCTTAAACGTCGCCGTTATACTGGTTGGGGACGTTTGTCTCGAAAATTGATTAATGGTATTAGGGATAAGCAATCTGGCAAAACAATATTAGATTTTTTGAAATCAGATGGTTTTGCCAATCGCAATTTTATGCAGCTGATCCATGATGATAGTTTGACATTTAAAGAAGACATTCAAAAAGCACAAGTGTCTGGACAAGGCGATAGTTTACATGAACATATTGCAAATTTAGCTGGTAGCCCTGCTATTAAAAAAGGTATTTTACAGACTGTAAAAGTTGTTGATGAATTGGTCAAAGTAATGGGGCGGCATAAGCCAGAAAATATCGTTATTGAAATGGCACGTGAAAATCAGACAACTCAAAAGGGCCAGAAAAATTCGCGAGAGCGTATGAAACGAATCGAAGAAGGTATCAAAGAATTAGGAAGTCAGATTCTTAAAGAGCATCCTGTTGAAAATACTCAATTGCAAAATGAAAAGCTCTATCTCTATTATCTCCAAAATGGAAGAGACATGTATGTGGACCAAGAATTAGATATTAATCGTTTAAGTGATTATGATGTCGATGCCATTGTTCCACAAAGTTTCCTTAAAGACGATTCAATAGACAATAAGGTCTTAACGCGTTCTGATAAAAATCGTGGTAAATCGGATAACGTTCCAAGTGAAGAAGTAGTCAAAAAGATGAAAAACTATTGGAGACAACTTCTAAACGCCAAGTTAATCACTCAACGTAAGTTTGATAATTTAACGAAAGCTGAACGTGGAGGTTTGAGTGAACTTGATAAAGCTGGTTTTATCAAACGCCAATTGGTTGAAACTCGCCAAATCACTAAGCATGTGGCACAAATTTTGGATAGTCGCATGAATACTAAATACGATGAAAATGATAAACTTATTCGAGAGGTTAAAGTGATTACCTTAAAATCTAAATTAGTTTCTGACTTCCGAAAAGATTTCCAATTCTATAAAGTACGTGAGATTAACAATTACCATCATGCCCATGATGCGTATCTAAATGCCGTCGTTGGAACTGCTTTGATTAAGAAATATCCAAAACTTGAATCGGAGTTTGTCTATGGTGATTATAAAGTTTATGATGTTCGTAAAATGATTGCTAAGTCTGAGCAAGAAATAGGCAAAGCAACCGCAAAATATTTCTTTTACTCTAATATCATGAACTTCTTCAAAACAGAAATTACACTTGCAAATGGAGAGATTCGCAAACGCCCTCTAATCGAAACTAATGGGGAAACTGGAGAAATTGTCTGGGATAAAGGGCGAGATTTTGCCACAGTGCGCAAAGTATTGTCCATGCCCCAAGTCAATATTGTCAAGAAAACAGAAGTACAGACAGGCGGATTCTCCAAGGAGTCAATTTTACCAAAAAGAAATTCGGACAAGCTTATTGCTCGTAAAAAAGACTGGGATCCAAAAAAATATGGTGGTTTTGATAGTCCAACGGTAGCTTATTCAGTCCTAGTGGTTGCTAAGGTGGAAAAAGGGAAATCGAAGAAGTTAAAATCCGTTAAAGAGTTACTAGGGATCACAATTATGGAAAGAAGTTCCTTTGAAAAAAATCCGATTGACTTTTTAGAAGCTAAAGGATATAAGGAAGTTAAAAAAGACTTAATCATTAAACTACCTAAATATAGTCTTTTTGAGTTAGAAAACGGTCGTAAACGGATGCTGGCTAGTGCCGGAGAATTACAAAAAGGAAATGAGCTGGCTCTGCCAAGCAAATATGTGAATTTTTTATATTTAGCTAGTCATTATGAAAAGTTGAAGGGTAGTCCAGAAGATAACGAACAAAAACAATTGTTTGTGGAGCAGCATAAGCATTATTTAGATGAGATTATTGAGCAAATCAGTGAATTTTCTAAGCGTGTTATTTTAGCAGATGCCAATTTAGATAAAGTTCTTAGTGCATATAACAAACATAGAGACAAACCAATACGTGAACAAGCAGAAAATATTATTCATTTATTTACGTTGACGAATCTTGGAGCTCCCGCTGCTTTTAAATATTTTGATACAACAATTGATCGTAAACGATATACGTCTACAAAAGAAGTTTTAGATGCCACTCTTATCCATCAATCCATCACTGGTCTTTATGAAACACGCATTGATTTGAGTCAGCTAGGAGGTGACTAA |
| *dCas9*_ZFP* | gene | ATGGATAAGAAATACTCAATAGGCTTAGCTATCGGCACAAATAGCGTCGGATGGGCGGTGATCACTGATGAATATAAGGTTCCGTCTAAAAAGTTCAAGGTTCTGGGAAATACAGACCGCCACAGTATCAAAAAAAATCTTATAGGGGCTCTTTTATTTGACAGTGGAGAGACAGCGGAAGCGACTCGTCTCAAACGGACAGCTCGTAGAAGGTATACACGTCGGAAGAATCGTATTTGTTATCTACAGGAGATTTTTTCAAATGAGATGGCGAAAGTAGATGATAGTTTCTTTCATCGACTTGAAGAGTCTTTTTTGGTGGAAGAAGACAAGAAGCATGAACGTCATCCTATTTTTGGAAATATAGTAGATGAAGTTGCTTATCATGAGAAATATCCAACTATCTATCATCTGCGAAAAAAATTGGTAGATTCTACTGATAAAGCGGATTTGCGCTTAATCTATTTGGCCTTAGCGCATATGATTAAGTTTCGTGGTCATTTTTTGATTGAGGGAGATTTAAATCCTGATAATAGTGATGTGGACAAACTATTTATCCAGTTGGTACAAACCTACAATCAATTATTTGAAGAAAACCCTATTAACGCAAGTGGAGTAGATGCTAAAGCGATTCTTTCTGCACGATTGAGTAAATCAAGACGATTAGAAAATCTCATTGCTCAGCTCCCCGGTGAGAAGAAAAATGGCTTATTTGGGAATCTCATTGCTTTGTCATTGGGTTTGACCCCTAATTTTAAATCAAATTTTGATTTGGCAGAAGATGCTAAATTACAGCTTTCAAAAGATACTTACGATGATGATTTAGATAATTTATTGGCGCAAATTGGAGATCAATATGCTGATTTGTTTTTGGCAGCTAAGAATTTATCAGATGCTATTTTACTTTCAGATATCCTAAGAGTAAATACTGAAATAACTAAGGCTCCCCTATCAGCTTCAATGATTAAACGCTACGATGAACATCATCAAGACTTGACTCTTTTAAAAGCTTTAGTTCGACAACAACTTCCAGAAAAGTATAAAGAAATCTTTTTTGATCAATCAAAAAACGGATATGCAGGTTATATTGATGGGGGAGCTAGCCAAGAAGAATTTTATAAATTTATCAAACCAATTTTAGAAAAAATGGATGGTACTGAGGAATTATTGGTGAAACTAAATCGTGAAGATTTGCTGCGCAAGCAACGGACCTTTGACAACGGCTCTATTCCCCATCAAATTCACTTGGGTGAGCTGCATGCTATTTTGAGAAGACAAGAAGACTTTTATCCATTTTTAAAAGACAATCGTGAGAAGATTGAAAAAATCTTGACTTTTCGAATTCCTTATTATGTTGGTCCATTGGCGCGTGGCAATAGTCGTTTTGCATGGATGACTCGGAAGTCTGAAGAAACAATTACCCCATGGAATTTTGAAGAAGTTGTCGATAAAGGTGCTTCAGCTCAATCATTTATTGAACGCATGACAAACTTTGATAAAAATCTTCCAAATGAAAAAGTACTACCAAAACATAGTTTGCTTTATGAGTATTTTACGGTTTATAACGAATTGACAAAGGTCAAATATGTTACTGAAGGAATGCGAAAACCAGCATTTCTTTCAGGTGAACAGAAGAAAGCCATTGTTGATTTACTCTTCAAAACAAATCGAAAAGTAACCGTTAAGCAATTAAAAGAAGATTATTTCAAAAAAATAGAATGTTTTGATAGTGTTGAAATTTCAGGAGTTGAAGATAGATTTAATGCTTCATTAGGTACCTACCATGATTTGCTAAAAATTATTAAAGATAAAGATTTTTTGGATAATGAAGAAAATGAAGATATCTTAGAGGATATTGTTTTAACATTGACCTTATTTGAAGATAGGGAGATGATTGAGGAAAGACTTAAAACATATGCTCACCTCTTTGATGATAAGGTGATGAAACAGCTTAAACGTCGCCGTTATACTGGTTGGGGACGTTTGTCTCGAAAATTGATTAATGGTATTAGGGATAAGCAATCTGGCAAAACAATATTAGATTTTTTGAAATCAGATGGTTTTGCCAATCGCAATTTTATGCAGCTGATCCATGATGATAGTTTGACATTTAAAGAAGACATTCAAAAAGCACAAGTGTCTGGACAAGGCGATAGTTTACATGAACATATTGCAAATTTAGCTGGTAGCCCTGCTATTAAAAAAGGTATTTTACAGACTGTAAAAGTTGTTGATGAATTGGTCAAAGTAATGGGGCGGCATAAGCCAGAAAATATCGTTATTGAAATGGCACGTGAAAATCAGACAACTCAAAAGGGCCAGAAAAATTCGCGAGAGCGTATGAAACGAATCGAAGAAGGTATCAAAGAATTAGGAAGTCAGATTCTTAAAGAGCATCCTGTTGAAAATACTCAATTGCAAAATGAAAAGCTCTATCTCTATTATCTCCAAAATGGAAGAGACATGTATGTGGACCAAGAATTAGATATTAATCGTTTAAGTGATTATGATGTCGATGCCATTGTTCCACAAAGTTTCCTTAAAGACGATTCAATAGACAATAAGGTCTTAACGCGTTCTGATAAAAATCGTGGTAAATCGGATAACGTTCCAAGTGAAGAAGTAGTCAAAAAGATGAAAAACTATTGGAGACAACTTCTAAACGCCAAGTTAATCACTCAACGTAAGTTTGATAATTTAACGAAAGCTGAACGTGGAGGTTTGAGTGAACTTGATAAAGCTGGTTTTATCAAACGCCAATTGGTTGAAACTCGCCAAATCACTAAGCATGTGGCACAAATTTTGGATAGTCGCATGAATACTAAATACGATGAAAATGATAAACTTATTCGAGAGGTTAAAGTGATTACCTTAAAATCTAAATTAGTTTCTGACTTCCGAAAAGATTTCCAATTCTATAAAGTACGTGAGATTAACAATTACCATCATGCCCATGATGCGTATCTAAATGCCGTCGTTGGAACTGCTTTGATTAAGAAATATCCAAAACTTGAATCGGAGTTTGTCTATGGTGATTATAAAGTTTATGATGTTCGTAAAATGATTGCTAAGTCTGAGCAAGAAATAGGCAAAGCAACCGCAAAATATTTCTTTTACTCTAATATCATGAACTTCTTCAAAACAGAAATTACACTTGCAAATGGAGAGATTCGCAAACGCCCTCTAATCGAAACTAATGGGGAAACTGGAGAAATTGTCTGGGATAAAGGGCGAGATTTTGCCACAGTGCGCAAAGTATTGTCCATGCCCCAAGTCAATATTGTCAAGAAAACAGAAGTACAGACAGGCGGATTCTCCAAGGAGTCAATTTTACCAAAAAGAAATTCGGACAAGCTTATTGCTCGTAAAAAAGACTGGGATCCAAAAAAATATGGTGGTTTTGATAGTCCAACGGTAGCTTATTCAGTCCTAGTGGTTGCTAAGGTGGAAAAAGGGAAATCGAAGAAGTTAAAATCCGTTAAAGAGTTACTAGGGATCACAATTATGGAAAGAAGTTCCTTTGAAAAAAATCCGATTGACTTTTTAGAAGCTAAAGGATATAAGGAAGTTAAAAAAGACTTAATCATTAAACTACCTAAATATAGTCTTTTTGAGTTAGAAAACGGTCGTAAACGGATGCTGGCTAGTGCCGGAGAATTACAAAAAGGAAATGAGCTGGCTCTGCCAAGCAAATATGTGAATTTTTTATATTTAGCTAGTCATTATGAAAAGTTGAAGGGTAGTCCAGAAGATAACGAACAAAAACAATTGTTTGTGGAGCAGCATAAGCATTATTTAGATGAGATTATTGAGCAAATCAGTGAATTTTCTAAGCGTGTTATTTTAGCAGATGCCAATTTAGATAAAGTTCTTAGTGCATATAACAAACATAGAGACAAACCAATACGTGAACAAGCAGAAAATATTATTCATTTATTTACGTTGACGAATCTTGGAGCTCCCGCTGCTTTTAAATATTTTGATACAACAATTGATCGTAAAAAGTATACGTCTACAAAAGAAGTTTTAGATGCCACTCTTATCCATCAATCCATCACTGGTCTTTATGAAACACGCATTGATTTGAGTCAGCTAGGAGGTGACGGCACCGGCGGGCCCAAGAAGAAGAGGAAGGTATACCCATACGATGTTCCTGACTATGCGGGCTATCCCTATGACGTCCCGGACTATGCAGGATCGTATCCTTATGACGTTCCAGATTACGCTGGATCCGCCGCTCCGGCAGCTAAGAAAAAGAAACTGGATTTCGAATCCGGAAAGCCCTATAAATGTCCTGAATGTGGCAAGTCCTTCTCGCGGAGCGACGACCTGACACGGCACCAACGTACGCACACTGGTGAGAAGCCATACGCGTGTCCTGTCGAGTCCTGTGACCGCCGCTTCAGTCAGAAGGGACACCTGACACGGCACATCCGCATTCACACAGGGCAAAAACCGTTTCAATGCCGCATCTGCATGAGGAACTTCAGCATCCGTAGCAGCCTGACACGGCACATCCGCACCCACACAGGAGAAAAGCCCTTCGCCTGTGACATCTGCGGCAGGAAGTTCGCGCTGAGCCACCACCTGACACGGCACACCAAGATCCACCTCCGTCAGAAAGACCCCGGGTAA |
| *dCas9*_HNH* | gene | ATGGATAAGAAATACTCAATAGGCTTAGCTATCGGCACAAATAGCGTCGGATGGGCGGTGATCACTGATGAATATAAGGTTCCGTCTAAAAAGTTCAAGGTTCTGGGAAATACAGACCGCCACAGTATCAAAAAAAATCTTATAGGGGCTCTTTTATTTGACAGTGGAGAGACAGCGGAAGCGACTCGTCTCAAACGGACAGCTCGTAGAAGGTATACACGTCGGAAGAATCGTATTTGTTATCTACAGGAGATTTTTTCAAATGAGATGGCGAAAGTAGATGATAGTTTCTTTCATCGACTTGAAGAGTCTTTTTTGGTGGAAGAAGACAAGAAGCATGAACGTCATCCTATTTTTGGAAATATAGTAGATGAAGTTGCTTATCATGAGAAATATCCAACTATCTATCATCTGCGAAAAAAATTGGTAGATTCTACTGATAAAGCGGATTTGCGCTTAATCTATTTGGCCTTAGCGCATATGATTAAGTTTCGTGGTCATTTTTTGATTGAGGGAGATTTAAATCCTGATAATAGTGATGTGGACAAACTATTTATCCAGTTGGTACAAACCTACAATCAATTATTTGAAGAAAACCCTATTAACGCAAGTGGAGTAGATGCTAAAGCGATTCTTTCTGCACGATTGAGTAAATCAAGACGATTAGAAAATCTCATTGCTCAGCTCCCCGGTGAGAAGAAAAATGGCTTATTTGGGAATCTCATTGCTTTGTCATTGGGTTTGACCCCTAATTTTAAATCAAATTTTGATTTGGCAGAAGATGCTAAATTACAGCTTTCAAAAGATACTTACGATGATGATTTAGATAATTTATTGGCGCAAATTGGAGATCAATATGCTGATTTGTTTTTGGCAGCTAAGAATTTATCAGATGCTATTTTACTTTCAGATATCCTAAGAGTAAATACTGAAATAACTAAGGCTCCCCTATCAGCTTCAATGATTAAACGCTACGATGAACATCATCAAGACTTGACTCTTTTAAAAGCTTTAGTTCGACAACAACTTCCAGAAAAGTATAAAGAAATCTTTTTTGATCAATCAAAAAACGGATATGCAGGTTATATTGATGGGGGAGCTAGCCAAGAAGAATTTTATAAATTTATCAAACCAATTTTAGAAAAAATGGATGGTACTGAGGAATTATTGGTGAAACTAAATCGTGAAGATTTGCTGCGCAAGCAACGGACCTTTGACAACGGCTCTATTCCCCATCAAATTCACTTGGGTGAGCTGCATGCTATTTTGAGAAGACAAGAAGACTTTTATCCATTTTTAAAAGACAATCGTGAGAAGATTGAAAAAATCTTGACTTTTCGAATTCCTTATTATGTTGGTCCATTGGCGCGTGGCAATAGTCGTTTTGCATGGATGACTCGGAAGTCTGAAGAAACAATTACCCCATGGAATTTTGAAGAAGTTGTCGATAAAGGTGCTTCAGCTCAATCATTTATTGAACGCATGACAAACTTTGATAAAAATCTTCCAAATGAAAAAGTACTACCAAAACATAGTTTGCTTTATGAGTATTTTACGGTTTATAACGAATTGACAAAGGTCAAATATGTTACTGAAGGAATGCGAAAACCAGCATTTCTTTCAGGTGAACAGAAGAAAGCCATTGTTGATTTACTCTTCAAAACAAATCGAAAAGTAACCGTTAAGCAATTAAAAGAAGATTATTTCAAAAAAATAGAATGTTTTGATAGTGTTGAAATTTCAGGAGTTGAAGATAGATTTAATGCTTCATTAGGTACCTACCATGATTTGCTAAAAATTATTAAAGATAAAGATTTTTTGGATAATGAAGAAAATGAAGATATCTTAGAGGATATTGTTTTAACATTGACCTTATTTGAAGATAGGGAGATGATTGAGGAAAGACTTAAAACATATGCTCACCTCTTTGATGATAAGGTGATGAAACAGCTTAAACGTCGCCGTTATACTGGTTGGGGACGTTTGTCTCGAAAATTGATTAATGGTATTAGGGATAAGCAATCTGGCAAAACAATATTAGATTTTTTGAAATCAGATGGTTTTGCCAATCGCAATTTTATGCAGCTGATCCATGATGATAGTTTGACATTTAAAGAAGACATTCAAAAAGCACAAGTGTCTGGACAAGGCGATAGTTTACATGAACATATTGCAAATTTAGCTGGTAGCCCTGCTATTAAAAAAGGTATTTTACAGACTGTAAAAGTTGTTGATGAATTGGTCAAAGTAATGGGGCGGCATAAGCCAGAAAATATCGTTATTGAAATGGCACGTGAAAATCAGGGAGGTTCAGGTGGATCGCGCCAATTGGTTGAAACTCGCCAAATCACTAAGCATGTGGCACAAATTTTGGATAGTCGCATGAATACTAAATACGATGAAAATGATAAACTTATTCGAGAGGTTAAAGTGATTACCTTAAAATCTAAATTAGTTTCTGACTTCCGAAAAGATTTCCAATTCTATAAAGTACGTGAGATTAACAATTACCATCATGCCCATGATGCGTATCTAAATGCCGTCGTTGGAACTGCTTTGATTAAGAAATATCCAAAACTTGAATCGGAGTTTGTCTATGGTGATTATAAAGTTTATGATGTTCGTAAAATGATTGCTAAGTCTGAGCAAGAAATAGGCAAAGCAACCGCAAAATATTTCTTTTACTCTAATATCATGAACTTCTTCAAAACAGAAATTACACTTGCAAATGGAGAGATTCGCAAACGCCCTCTAATCGAAACTAATGGGGAAACTGGAGAAATTGTCTGGGATAAAGGGCGAGATTTTGCCACAGTGCGCAAAGTATTGTCCATGCCCCAAGTCAATATTGTCAAGAAAACAGAAGTACAGACAGGCGGATTCTCCAAGGAGTCAATTTTACCAAAAAGAAATTCGGACAAGCTTATTGCTCGTAAAAAAGACTGGGATCCAAAAAAATATGGTGGTTTTGATAGTCCAACGGTAGCTTATTCAGTCCTAGTGGTTGCTAAGGTGGAAAAAGGGAAATCGAAGAAGTTAAAATCCGTTAAAGAGTTACTAGGGATCACAATTATGGAAAGAAGTTCCTTTGAAAAAAATCCGATTGACTTTTTAGAAGCTAAAGGATATAAGGAAGTTAAAAAAGACTTAATCATTAAACTACCTAAATATAGTCTTTTTGAGTTAGAAAACGGTCGTAAACGGATGCTGGCTAGTGCCGGAGAATTACAAAAAGGAAATGAGCTGGCTCTGCCAAGCAAATATGTGAATTTTTTATATTTAGCTAGTCATTATGAAAAGTTGAAGGGTAGTCCAGAAGATAACGAACAAAAACAATTGTTTGTGGAGCAGCATAAGCATTATTTAGATGAGATTATTGAGCAAATCAGTGAATTTTCTAAGCGTGTTATTTTAGCAGATGCCAATTTAGATAAAGTTCTTAGTGCATATAACAAACATAGAGACAAACCAATACGTGAACAAGCAGAAAATATTATTCATTTATTTACGTTGACGAATCTTGGAGCTCCCGCTGCTTTTAAATATTTTGATACAACAATTGATCGTAAAAAGTATACGTCTACAAAAGAAGTTTTAGATGCCACTCTTATCCATCAATCCATCACTGGTCTTTATGAAACACGCATTGATTTGAGTCAGCTAGGAGGTGACGGCACCGGCGGGCCCAAGAAGAAGAGGAAGGTATACCCATACGATGTTCCTGACTATGCGGGCTATCCCTATGACGTCCCGGACTATGCAGGATCGTATCCTTATGACGTTCCAGATTACGCTGGATCCGCCGCTCCGGCAGCTAAGAAAAAGAAACTGGATTTCGAATCCGGAAAGCCCTATAAATGTCCTGAATGTGGCAAGTCCTTCTCGCGGAGCGACGACCTGACACGGCACCAACGTACGCACACTGGTGAGAAGCCATACGCGTGTCCTGTCGAGTCCTGTGACCGCCGCTTCAGTCAGAAGGGACACCTGACACGGCACATCCGCATTCACACAGGGCAAAAACCGTTTCAATGCCGCATCTGCATGAGGAACTTCAGCATCCGTAGCAGCCTGACACGGCACATCCGCACCCACACAGGAGAAAAGCCCTTCGCCTGTGACATCTGCGGCAGGAAGTTCGCGCTGAGCCACCACCTGACACGGCACACCAAGATCCACCTCCGTCAGAAAGACCCCGGGTAA |
| *dCas9*_HNH-L88* | gene | ATGGATAAGAAATACTCAATAGGCTTAGCTATCGGCACAAATAGCGTCGGATGGGCGGTGATCACTGATGAATATAAGGTTCCGTCTAAAAAGTTCAAGGTTCTGGGAAATACAGACCGCCACAGTATCAAAAAAAATCTTATAGGGGCTCTTTTATTTGACAGTGGAGAGACAGCGGAAGCGACTCGTCTCAAACGGACAGCTCGTAGAAGGTATACACGTCGGAAGAATCGTATTTGTTATCTACAGGAGATTTTTTCAAATGAGATGGCGAAAGTAGATGATAGTTTCTTTCATCGACTTGAAGAGTCTTTTTTGGTGGAAGAAGACAAGAAGCATGAACGTCATCCTATTTTTGGAAATATAGTAGATGAAGTTGCTTATCATGAGAAATATCCAACTATCTATCATCTGCGAAAAAAATTGGTAGATTCTACTGATAAAGCGGATTTGCGCTTAATCTATTTGGCCTTAGCGCATATGATTAAGTTTCGTGGTCATTTTTTGATTGAGGGAGATTTAAATCCTGATAATAGTGATGTGGACAAACTATTTATCCAGTTGGTACAAACCTACAATCAATTATTTGAAGAAAACCCTATTAACGCAAGTGGAGTAGATGCTAAAGCGATTCTTTCTGCACGATTGAGTAAATCAAGACGATTAGAAAATCTCATTGCTCAGCTCCCCGGTGAGAAGAAAAATGGCTTATTTGGGAATCTCATTGCTTTGTCATTGGGTTTGACCCCTAATTTTAAATCAAATTTTGATTTGGCAGAAGATGCTAAATTACAGCTTTCAAAAGATACTTACGATGATGATTTAGATAATTTATTGGCGCAAATTGGAGATCAATATGCTGATTTGTTTTTGGCAGCTAAGAATTTATCAGATGCTATTTTACTTTCAGATATCCTAAGAGTAAATACTGAAATAACTAAGGCTCCCCTATCAGCTTCAATGATTAAACGCTACGATGAACATCATCAAGACTTGACTCTTTTAAAAGCTTTAGTTCGACAACAACTTCCAGAAAAGTATAAAGAAATCTTTTTTGATCAATCAAAAAACGGATATGCAGGTTATATTGATGGGGGAGCTAGCCAAGAAGAATTTTATAAATTTATCAAACCAATTTTAGAAAAAATGGATGGTACTGAGGAATTATTGGTGAAACTAAATCGTGAAGATTTGCTGCGCAAGCAACGGACCTTTGACAACGGCTCTATTCCCCATCAAATTCACTTGGGTGAGCTGCATGCTATTTTGAGAAGACAAGAAGACTTTTATCCATTTTTAAAAGACAATCGTGAGAAGATTGAAAAAATCTTGACTTTTCGAATTCCTTATTATGTTGGTCCATTGGCGCGTGGCAATAGTCGTTTTGCATGGATGACTCGGAAGTCTGAAGAAACAATTACCCCATGGAATTTTGAAGAAGTTGTCGATAAAGGTGCTTCAGCTCAATCATTTATTGAACGCATGACAAACTTTGATAAAAATCTTCCAAATGAAAAAGTACTACCAAAACATAGTTTGCTTTATGAGTATTTTACGGTTTATAACGAATTGACAAAGGTCAAATATGTTACTGAAGGAATGCGAAAACCAGCATTTCTTTCAGGTGAACAGAAGAAAGCCATTGTTGATTTACTCTTCAAAACAAATCGAAAAGTAACCGTTAAGCAATTAAAAGAAGATTATTTCAAAAAAATAGAATGTTTTGATAGTGTTGAAATTTCAGGAGTTGAAGATAGATTTAATGCTTCATTAGGTACCTACCATGATTTGCTAAAAATTATTAAAGATAAAGATTTTTTGGATAATGAAGAAAATGAAGATATCTTAGAGGATATTGTTTTAACATTGACCTTATTTGAAGATAGGGAGATGATTGAGGAAAGACTTAAAACATATGCTCACCTCTTTGATGATAAGGTGATGAAACAGCTTAAACGTCGCCGTTATACTGGTTGGGGACGTTTGTCTCGAAAATTGATTAATGGTATTAGGGATAAGCAATCTGGCAAAACAATATTAGATTTTTTGAAATCAGATGGTTTTGCCAATCGCAATTTTATGCAGCTGATCCATGATGATAGTTTGACATTTAAAGAAGACATTCAAAAAGCACAAGTGTCTGGACAAGGCGATAGTTTACATGAACATATTGCAAATTTAGCTGGTAGCCCTGCTATTAAAAAAGGTATTTTACAGACTGTAAAAGTTGTTGATGAATTGGTCAAAGTAATGGGGCGGCATAAGCCAGAAAATATCGTTATTGAAATGGCACGTGAAAATCAGGGAGGTTCAGGTGGATCGCGCCAATTGGTTGAAACTCGCCAAATCACTAAGCATGTGGCACAAATTTTGGATAGTCGCATGAATACTAAATACGATGAAAATGATAAACTTATTCGAGAGGTTAAAGTGATTACCTTAAAATCTAAATTAGTTTCTGACTTCCGAAAAGATTTCCAATTCTATAAAGTACGTGAGATTAACAATTACCATCATGCCCATGATGCGTATCTAAATGCCGTCGTTGGAACTGCTTTGATTAAGAAATATCCAAAACTTGAATCGGAGTTTGTCTATGGTGATTATAAAGTTTATGATGTTCGTAAAATGATTGCTAAGTCTGAGCAAGAAATAGGCAAAGCAACCGCAAAATATTTCTTTTACTCTAATATCATGAACTTCTTCAAAACAGAAATTACACTTGCAAATGGAGAGATTCGCAAACGCCCTCTAATCGAAACTAATGGGGAAACTGGAGAAATTGTCTGGGATAAAGGGCGAGATTTTGCCACAGTGCGCAAAGTATTGTCCATGCCCCAAGTCAATATTGTCAAGAAAACAGAAGTACAGACAGGCGGATTCTCCAAGGAGTCAATTTTACCAAAAAGAAATTCGGACAAGCTTATTGCTCGTAAAAAAGACTGGGATCCAAAAAAATATGGTGGTTTTGATAGTCCAACGGTAGCTTATTCAGTCCTAGTGGTTGCTAAGGTGGAAAAAGGGAAATCGAAGAAGTTAAAATCCGTTAAAGAGTTACTAGGGATCACAATTATGGAAAGAAGTTCCTTTGAAAAAAATCCGATTGACTTTTTAGAAGCTAAAGGATATAAGGAAGTTAAAAAAGACTTAATCATTAAACTACCTAAATATAGTCTTTTTGAGTTAGAAAACGGTCGTAAACGGATGCTGGCTAGTGCCGGAGAATTACAAAAAGGAAATGAGCTGGCTCTGCCAAGCAAATATGTGAATTTTTTATATTTAGCTAGTCATTATGAAAAGTTGAAGGGTAGTCCAGAAGATAACGAACAAAAACAATTGTTTGTGGAGCAGCATAAGCATTATTTAGATGAGATTATTGAGCAAATCAGTGAATTTTCTAAGCGTGTTATTTTAGCAGATGCCAATTTAGATAAAGTTCTTAGTGCATATAACAAACATAGAGACAAACCAATACGTGAACAAGCAGAAAATATTATTCATTTATTTACGTTGACGAATCTTGGAGCTCCCGCTGCTTTTAAATATTTTGATACAACAATTGATCGTAAAAAGTATACGTCTACAAAAGAAGTTTTAGATGCCACTCTTATCCATCAATCCATCACTGGTCTTTATGAAACACGCATTGATTTGAGTCAGCTAGGAGGTGACGGCACCGGCGGGCCCAAGAAGAAGAGGAAGGTATACCCATACGATGTTCCTGACTATGCGGGCTATCCCTATGACGTCCCGGACTATGCAGGATCGTATCCTTATGACGTTCCAGATTACGCTGGATCCGCCGCTCCGGCAGCTAAGAAAAAGAAACTGGATTACCCGTATGACGTACCTGATTACGCTGGTTATCCCTATGATGTCCCGGACTACGCTGGCTCGTACCCTTATGATGTACCTGACTACGCTTTCGAATCCGGAAAGCCCTATAAATGTCCTGAATGTGGCAAGTCCTTCTCGCGGAGCGACGACCTGACACGGCACCAACGTACGCACACTGGTGAGAAGCCATACGCGTGTCCTGTCGAGTCCTGTGACCGCCGCTTCAGTCAGAAGGGACACCTGACACGGCACATCCGCATTCACACAGGGCAAAAACCGTTTCAATGCCGCATCTGCATGAGGAACTTCAGCATCCGTAGCAGCCTGACACGGCACATCCGCACCCACACAGGAGAAAAGCCCTTCGCCTGTGACATCTGCGGCAGGAAGTTCGCGCTGAGCCACCACCTGACACGGCACACCAAGATCCACCTCCGTCAGAAAGACCCCGGGTAA |
| *dCas9*_PhlF* | gene | ATGGATAAGAAATACTCAATAGGCTTAGCTATCGGCACAAATAGCGTCGGATGGGCGGTGATCACTGATGAATATAAGGTTCCGTCTAAAAAGTTCAAGGTTCTGGGAAATACAGACCGCCACAGTATCAAAAAAAATCTTATAGGGGCTCTTTTATTTGACAGTGGAGAGACAGCGGAAGCGACTCGTCTCAAACGGACAGCTCGTAGAAGGTATACACGTCGGAAGAATCGTATTTGTTATCTACAGGAGATTTTTTCAAATGAGATGGCGAAAGTAGATGATAGTTTCTTTCATCGACTTGAAGAGTCTTTTTTGGTGGAAGAAGACAAGAAGCATGAACGTCATCCTATTTTTGGAAATATAGTAGATGAAGTTGCTTATCATGAGAAATATCCAACTATCTATCATCTGCGAAAAAAATTGGTAGATTCTACTGATAAAGCGGATTTGCGCTTAATCTATTTGGCCTTAGCGCATATGATTAAGTTTCGTGGTCATTTTTTGATTGAGGGAGATTTAAATCCTGATAATAGTGATGTGGACAAACTATTTATCCAGTTGGTACAAACCTACAATCAATTATTTGAAGAAAACCCTATTAACGCAAGTGGAGTAGATGCTAAAGCGATTCTTTCTGCACGATTGAGTAAATCAAGACGATTAGAAAATCTCATTGCTCAGCTCCCCGGTGAGAAGAAAAATGGCTTATTTGGGAATCTCATTGCTTTGTCATTGGGTTTGACCCCTAATTTTAAATCAAATTTTGATTTGGCAGAAGATGCTAAATTACAGCTTTCAAAAGATACTTACGATGATGATTTAGATAATTTATTGGCGCAAATTGGAGATCAATATGCTGATTTGTTTTTGGCAGCTAAGAATTTATCAGATGCTATTTTACTTTCAGATATCCTAAGAGTAAATACTGAAATAACTAAGGCTCCCCTATCAGCTTCAATGATTAAACGCTACGATGAACATCATCAAGACTTGACTCTTTTAAAAGCTTTAGTTCGACAACAACTTCCAGAAAAGTATAAAGAAATCTTTTTTGATCAATCAAAAAACGGATATGCAGGTTATATTGATGGGGGAGCTAGCCAAGAAGAATTTTATAAATTTATCAAACCAATTTTAGAAAAAATGGATGGTACTGAGGAATTATTGGTGAAACTAAATCGTGAAGATTTGCTGCGCAAGCAACGGACCTTTGACAACGGCTCTATTCCCCATCAAATTCACTTGGGTGAGCTGCATGCTATTTTGAGAAGACAAGAAGACTTTTATCCATTTTTAAAAGACAATCGTGAGAAGATTGAAAAAATCTTGACTTTTCGAATTCCTTATTATGTTGGTCCATTGGCGCGTGGCAATAGTCGTTTTGCATGGATGACTCGGAAGTCTGAAGAAACAATTACCCCATGGAATTTTGAAGAAGTTGTCGATAAAGGTGCTTCAGCTCAATCATTTATTGAACGCATGACAAACTTTGATAAAAATCTTCCAAATGAAAAAGTACTACCAAAACATAGTTTGCTTTATGAGTATTTTACGGTTTATAACGAATTGACAAAGGTCAAATATGTTACTGAAGGAATGCGAAAACCAGCATTTCTTTCAGGTGAACAGAAGAAAGCCATTGTTGATTTACTCTTCAAAACAAATCGAAAAGTAACCGTTAAGCAATTAAAAGAAGATTATTTCAAAAAAATAGAATGTTTTGATAGTGTTGAAATTTCAGGAGTTGAAGATAGATTTAATGCTTCATTAGGTACCTACCATGATTTGCTAAAAATTATTAAAGATAAAGATTTTTTGGATAATGAAGAAAATGAAGATATCTTAGAGGATATTGTTTTAACATTGACCTTATTTGAAGATAGGGAGATGATTGAGGAAAGACTTAAAACATATGCTCACCTCTTTGATGATAAGGTGATGAAACAGCTTAAACGTCGCCGTTATACTGGTTGGGGACGTTTGTCTCGAAAATTGATTAATGGTATTAGGGATAAGCAATCTGGCAAAACAATATTAGATTTTTTGAAATCAGATGGTTTTGCCAATCGCAATTTTATGCAGCTGATCCATGATGATAGTTTGACATTTAAAGAAGACATTCAAAAAGCACAAGTGTCTGGACAAGGCGATAGTTTACATGAACATATTGCAAATTTAGCTGGTAGCCCTGCTATTAAAAAAGGTATTTTACAGACTGTAAAAGTTGTTGATGAATTGGTCAAAGTAATGGGGCGGCATAAGCCAGAAAATATCGTTATTGAAATGGCACGTGAAAATCAGGGAGGTTCAGGTGGATCGCGCCAATTGGTTGAAACTCGCCAAATCACTAAGCATGTGGCACAAATTTTGGATAGTCGCATGAATACTAAATACGATGAAAATGATAAACTTATTCGAGAGGTTAAAGTGATTACCTTAAAATCTAAATTAGTTTCTGACTTCCGAAAAGATTTCCAATTCTATAAAGTACGTGAGATTAACAATTACCATCATGCCCATGATGCGTATCTAAATGCCGTCGTTGGAACTGCTTTGATTAAGAAATATCCAAAACTTGAATCGGAGTTTGTCTATGGTGATTATAAAGTTTATGATGTTCGTAAAATGATTGCTAAGTCTGAGCAAGAAATAGGCAAAGCAACCGCAAAATATTTCTTTTACTCTAATATCATGAACTTCTTCAAAACAGAAATTACACTTGCAAATGGAGAGATTCGCAAACGCCCTCTAATCGAAACTAATGGGGAAACTGGAGAAATTGTCTGGGATAAAGGGCGAGATTTTGCCACAGTGCGCAAAGTATTGTCCATGCCCCAAGTCAATATTGTCAAGAAAACAGAAGTACAGACAGGCGGATTCTCCAAGGAGTCAATTTTACCAAAAAGAAATTCGGACAAGCTTATTGCTCGTAAAAAAGACTGGGATCCAAAAAAATATGGTGGTTTTGATAGTCCAACGGTAGCTTATTCAGTCCTAGTGGTTGCTAAGGTGGAAAAAGGGAAATCGAAGAAGTTAAAATCCGTTAAAGAGTTACTAGGGATCACAATTATGGAAAGAAGTTCCTTTGAAAAAAATCCGATTGACTTTTTAGAAGCTAAAGGATATAAGGAAGTTAAAAAAGACTTAATCATTAAACTACCTAAATATAGTCTTTTTGAGTTAGAAAACGGTCGTAAACGGATGCTGGCTAGTGCCGGAGAATTACAAAAAGGAAATGAGCTGGCTCTGCCAAGCAAATATGTGAATTTTTTATATTTAGCTAGTCATTATGAAAAGTTGAAGGGTAGTCCAGAAGATAACGAACAAAAACAATTGTTTGTGGAGCAGCATAAGCATTATTTAGATGAGATTATTGAGCAAATCAGTGAATTTTCTAAGCGTGTTATTTTAGCAGATGCCAATTTAGATAAAGTTCTTAGTGCATATAACAAACATAGAGACAAACCAATACGTGAACAAGCAGAAAATATTATTCATTTATTTACGTTGACGAATCTTGGAGCTCCCGCTGCTTTTAAATATTTTGATACAACAATTGATCGTAAAAAGTATACGTCTACAAAAGAAGTTTTAGATGCCACTCTTATCCATCAATCCATCACTGGTCTTTATGAAACACGCATTGATTTGAGTCAGCTAGGAGGTGACGGCACCGGCGGGCCCAAGAAGAAGAGGAAGGTATACCCATACGATGTTCCTGACTATGCGGGCTATCCCTATGACGTCCCGGACTATGCAGGATCGTATCCTTATGACGTTCCAGATTACGCTGGATCCGCCGCTCCGGCAGCTAAGAAAAAGAAACTGGATTACCCGTATGACGTACCTGATTACGCTGGTTATCCCTATGATGTCCCGGACTACGCTGGCTCGTACCCTTATGATGTACCTGACTACGCTTTCGAATCCGGAGCACGTACCCCGAGCCGTAGCAGCATTGGTAGCCTGCGTAGTCCGCATACCCATAAAGCAATTCTGACCAGCACCATTGAAATCCTGAAAGAATGTGGTTATAGCGGTCTGAGCATTGAAAGCGTTGCACGTCGTGCCGGTGCAAGCAAACCGACCATTTATCGTTGGTGGACCAATAAAGCAGCACTGATTGCCGAAGTGTATGAAAATGAAAGCGAACAGGTGCGTAAATTTCCGGATCTGGGTAGCTTTAAAGCCGATCTGGATTTTCTGCTGCGTAATCTGTGGAAAGTTTGGCGTGAAACCATTTGTGGTGAAGCATTTCGTTGTGTTATTGCAGAAGCACAGCTGGACCCTGCAACCCTGACCCAGCTGAAAGATCAGTTTATGGAACGTCGTCGTGAGATGCCGAAAAAACTGGTTGAAAATGCCATTAGCAATGGTGAACTGCCGAAAGATACCAATCGTGAACTGCTGCTGGATATGATTTTTGGTTTTTGTTGGTATCGCCTGCTGACCGAACAGCTGACCGTTGAACAGGATATTGAAGAATTTACCTTCCTGCTGATTAATGGTGTTTGTCCGGGTACACAGCGTTAA |
| *rfp* | gene | ATGGCTTCCTCCGAAGACGTTATCAAAGAGTTCATGCGTTTCAAAGTTCGTATGGAAGGTTCCGTTAACGGTCACGAGTTCGAAATCGAAGGTGAAGGTGAAGGTCGTCCGTACGAAGGTACCCAGACCGCTAAACTGAAAGTTACCAAAGGTGGTCCGCTGCCGTTCGCTTGGGACATCCTGTCCCCGCAGTTCCAGTACGGTTCCAAAGCTTACGTTAAACACCCGGCTGACATCCCGGACTACCTGAAACTGTCCTTCCCGGAAGGTTTCAAATGGGAACGTGTTATGAACTTCGAAGACGGTGGTGTTGTTACCGTTACCCAGGACTCCTCCCTGCAAGACGGTGAGTTCATCTACAAAGTTAAACTGCGTGGTACCAACTTCCCGTCCGACGGTCCGGTTATGCAGAAAAAAACCATGGGTTGGGAAGCTTCCACCGAACGTATGTACCCGGAAGACGGTGCTCTGAAAGGTGAAATCAAAATGCGTCTGAAACTGAAAGACGGTGGTCACTACGACGCTGAAGTTAAAACCACCTACATGGCTAAAAAACCGGTTCAGCTGCCGGGTGCTTACAAAACCGACATCAAACTGGACATCACCTCCCACAACGAAGACTACACCATCGTTGAACAGTACGAACGTGCTGAAGGTCGTCACTCCACCGGTGCTTAATAA |
| *lacI* | gene | ATGAAACCAGTAACGTTATACGATGTCGCAGAGTATGCCGGTGTCTCTTATCAGACCGTTTCCCGCGTGGTGAACCAGGCCAGCCACGTTTCTGCGAAAACGCGGGAAAAAGTGGAAGCGGCGATGGCGGAGCTGAATTACATTCCCAACCGCGTGGCACAACAACTGGCGGGCAAACAGTCGTTGCTGATTGGCGTTGCCACCTCCAGTCTGGCCCTGCACGCGCCGTCGCAAATTGTCGCGGCGATTAAATCTCGCGCCGATCAACTGGGTGCCAGCGTGGTGGTGTCGATGGTAGAACGAAGCGGCGTCGAAGCCTGTAAAGCGGCGGTGCACAATCTTCTCGCGCAACGCGTCAGTGGGCTGATCATTAACTATCCGCTGGATGACCAGGATGCCATTGCTGTGGAAGCTGCCTGCACTAATGTTCCGGCGTTATTTCTTGATGTCTCTGACCAGACACCCATCAACAGTATTATTTTCTCCCATGAGGACGGTACGCGACTGGGCGTGGAGCATCTGGTCGCATTGGGTCACCAGCAAATCGCGCTGTTAGCGGGCCCATTAAGTTCTGTCTCGGCGCGTCTGCGTCTGGCTGGCTGGCATAAATATCTCACTCGCAATCAAATTCAGCCGATAGCGGAACGGGAAGGCGACTGGAGTGCCATGTCCGGTTTTCAACAAACCATGCAAATGCTGAATGAGGGCATCGTTCCCACTGCGATGCTGGTTGCCAACGATCAGATGGCGCTGGGCGCAATGCGCGCCATTACCGAGTCCGGGCTGCGCGTTGGTGCGGATATCTCGGTAGTGGGATACGACGATACCGAAGATAGCTCATGTTATATCCCGCCGTTAACCACCATCAAACAGGATTTTCGCCTGCTGGGGCAAACCAGCGTGGACCGCTTGCTGCAACTCTCTCAGGGCCAGGCGGTGAAGGGCAATCAGCTGTTGCCAGTCTCACTGGTGAAAAGAAAAACCACCCTGGCGCCCAATACGCAAACCGCCTCTCCCCGCGCGTTGGCCGATTCATTAATGCAGCTGGCACGACAGGTTTCCCGACTGGAAAGCGGGCAGTGA |
| *tetR* | gene | ATGTCCAGATTAGATAAAAGTAAAGTGATTAACAGCGCATTAGAGCTGCTTAATGAGGTCGGAATCGAAGGTTTAACAACCCGTAAACTCGCCCAGAAGCTAGGTGTAGAGCAGCCTACATTGTATTGGCATGTAAAAAATAAGCGGGCTTTGCTCGACGCCTTAGCCATTGAGATGTTAGATAGGCACCATACTCACTTTTGCCCTTTAGAAGGGGAAAGCTGGCAAGATTTTTTACGTAATAACGCTAAAAGTTTTAGATGTGCTTTACTAAGTCATCGCGATGGAGCAAAAGTACATTTAGGTACACGGCCTACAGAAAAACAGTATGAAACTCTCGAAAATCAATTAGCCTTTTTATGCCAACAAGGTTTTTCACTAGAGAATGCATTATATGCACTCAGCGCTGTGGGGCATTTTACTTTAGGTTGCGTATTGGAAGATCAAGAGCATCAAGTCGCTAAAGAAGAAAGGGAAACACCTACTACTGATAGTATGCCGCCATTATTACGACAAGCTATCGAATTATTTGATCACCAAGGTGCAGAGCCAGCCTTCTTATTCGGCCTTGAATTGATCATATGCGGATTAGAAAAACAACTTAAATGTGAAAGTGGGTCCTAA |
| *vanR(1)* | gene | ATGGACATGCCTCGTATTAAACCGGGTCAGCGTGTTATGATGGCACTGCGTAAAATGATTGCAAGCGGTGAAATCAAAAGTGGTGAACGTATTGCAGAAATTCCGACCGCAGCAGCACTGGGTGTTAGCCGTATGCCGGTTCGTATCGCACTGCGTTCACTGGAACAAGAAGGTCTGGTTGTTCGTCTGGGTGCACGTGGTTATGCAGCCCGTGGTGTTAGCAGCGATCAGATTCGTGATGCAATTGAAGTTCGTGGTGTTCTGGAAGGTTTTGCAGCACGTCGTCTGGCAGAACGTGGTATGACCGCAGAAACCCATGCACGTTTTGTTGTACTGATTGCAGAAGGTGAAGCACTGTTTGCAGCCGGTCGCCTGAATGGTGAAGATCTGGATCGTTATGCCGCATATAATCAGGCATTTCATGATACCCTGGTTAGCGCAGCAGGTAATGGTGCAGTTGAAAGCGCACTGGCACGTAATGGTTTTGAACCGTTTGCAGCAGCCGGTGCACTGGCCCTGGATCTGATGGACCTGTCTGCCGAATATGAACATCTGCTGGCAGCACATCGTCAGCATCAGGCAGTTCTGGATGCAGTTAGCTGTGGTGATGCCGAAGGTGCAGAACGTATTATGCGTGATCATGCACTGGCAGCAATTCGTAATGCAAAAGTTTTTGAAGCAGCAGCAAGCGCAGGCGCACCGCTGGGTGCAGCATGGTCAATTCGTGCAGATTGA |
| *betI(1)* | gene | ATGCCGAAACTGGGTATGCAGAGCATTCGTCGTCGTCAGCTGATTGATGCAACCCTGGAAGCAATTAATGAAGTTGGTATGCATGATGCAACCATTGCACAGATTGCACGTCGTGCCGGTGTTAGCACCGGTATTATTAGCCATTATTTCCGCGATAAAAACGGTCTACTGGAAGCAACCATGCGTGATATTACCAGCCAGCTGCGTGATGCAGTTCTGAATCGTCTGCATGCACTGCCGCAGGGTAGCGCAGAACAGCGTCTGCAGGCAATTGTTGGTGGTAATTTTGATGAAACCCAGGTTAGCAGCGCAGCAATGAAAGCATGGCTGGCATTTTGGGCAATCAGCATGCATCAGCCGATGCTGTATCGTCTGCAGCAGGTTAGCAGTCGTCGTCTGCTGAGCAATCTGGTTAGCGAATTTCGTCGTGAACTGCCTCGTGAACAGGCACAAGAGGCAGGTTATGGTCTGGCAGCACTGATTGATGGTCTGTGGCTGCGTGCAGCACTGAGCGGTAAACCGCTGGATAAAACCCGTGCAAATAGCCTGACCCGTCATTTTATCACCCAGCATCTGCCGACCGATTGA |
| *luxR* | gene | ATGAAAAACATAAATGCCGACGACACATACAGAATAATTAATAAAATTAAAGCTTGTAGAAGCAATAATGATATTAATCAATGCTTATCTGATATGACTAAAATGGTACATTGTGAATATTATTTACTCGCGATCATTTATCCTCATTCTATGGTTAAATCTGATATTTCAATCCTAGATAATTACCCTAAAAAATGGAGGCAATATTATGATGACGCTAATTTAATAAAATATGATCCTATAGTAGATTATTCTAACTCCAATCATTCACCAATTAATTGGAATATATTTGAAAACAATGCTGTAAATAAAAAATCTCCAAATGTAATTAAAGAAGCGAAAACATCAGGTCTTATCACTGGGTTTAGTTTCCCTATTCATACGGCTAACAATGGCTTCGGAATGCTTAGTTTTGCACATTCAGAAAAAGACAACTATATAGATAGTTTATTTTTACATGCGTGTATGAACATACCATTAATTGTTCCTTCTCTAGTTGATAATTATCGAAAAATAAATATAGCAAATAATAAATCAAACAACGATTTAACCAAAAGAGAAAAAGAATGTTTAGCGTGGGCATGCGAAGGAAAAAGCTCTTGGGATATTTCAAAAATATTAGGTTGCAGTGAGCGTACTGTCACTTTCCATTTAACCAATGCGCAAATGAAACTCAATACAACAAACCGCTGCCAAAGTATTTCTAAAGCAATTTTAACAGGAGCAATTGATTGCCCATACTTTAAAAATTAA |
| T1 | Terminator | AAAAAAAAAAAAGGCCTCCCAAATCGGGGGGCCTTTTTTATTGATAACAAAA |
| T2 | Terminator | CTCGGTACCAAATTCCAGAAAAGAGACGCTTAACAGCGTCTTTTTTCGTTTTGGTCC |
| T3 | Terminator | CCAATTATTGAAGGCCGCTAACGCGGCCTTTTTTTGTTTCTGGTCTCCC |
| T4 | Terminator | CCAATTATTGAAGGCCTCCCAAATCGGGGGGCCTTTTTTATTGATAACAAAA |
| T5 | Terminator | CTCGGTACCAAAAAAAAAAAAAAAGACGCTGAAAAGCGTCTTTTTTCGTTTTGGTCC |
| T6 | Terminator | CTCGGTACCAAAAAAAAAAAAAAAGACGCTGAAAAGCGTCTTTTTTTTTTTTGGTCC |
| T7 | Terminator | CTCGGTACCAAACCAATTATTGAAGACGCTGAAAAGCGTCTTTTTTCGTTTTGGTCC |
| T8 | Terminator | CTCGGTACCAAATTCCAGAAAAGAGACGCTTTTAGAGCGTCTTTTTTCGTTTTGGTCC |
| T9 | Terminator | CTCGGTACCAAATTCCAGAAAAGAGACGCTGAAAAGCGTCTTTTTTTTTTTTGGTCC |
| T10 | Terminator | GACGAACAATAAGGCCTCCCTAACGGGGGGCCTTTTTTATTGATAACAAAA |
| T11 | Terminator | GACGAACAATAAGGCCTCCCGAAAGGGGGGCCTTTTTTATTGATAACAAAA |
| T12 | Terminator | TCTAACTAAAAACACCCTAACGGGTGTTTTTTTGTTTCTGGTCTGCC |
| T13 | Terminator | CCAATTATTGAACACCCTTCGGGGTGTTTTTTTGTTTCTGGTCTCCC |
| T14 | Terminator | CCAATTATTGAAGACGCTTAACAGCGTCTTTTTTTGTTTCTGGTCTCCC |
| T15 | Terminator | TTTTCGAAAAAACACCCTAACGGGTGTTTTTTTGTTTCTGGTCTCCC |
| T16 | Terminator | CTCGGTACCAAATCTAACTAAAAAGACGCTGAAAAGCGTCTTTTTTCGTTTTGGTCC |
| L3S2P55 | Terminator | CTCGGTACCAAAGACGAACAATAAGACGCTGAAAAGCGTCTTTTTTCGTTTTGGTCC |
| L3S2P53 | Terminator | CTCGGTACCAAACCAATTATTGAAGACGCTGAAAAGCGTCTTTTTTCGTTTTGGTCC |
| L3S2P11 | Terminator | CTCGGTACCAAATTCCAGAAAAGAGACGCTTTCGAGCGTCTTTTTTCGTTTTGGTCC |
| L3S2P44 | Terminator | CTCGGTACCAAACCAATTATTGAAGACGCTGAAAAGCGTCTTTTTTTGTTTCGGTCC |
| ECK120033737 | Terminator | GGAAACACAGAAAAAAGCCCGCACCTGACAGTGCGGGCTTTTTTTTTCGACCAAAGG |
| B0010* | Terminator | CCAGGCATCAAATAAAACGAAAGGCTCAGTCGAAAGACTGGGCCTTTCGTTTTATCTGTTGTTTGTCGGTGAACGCTCTCTACTAGAGTCACACTGGCTCACCTTCGGGTGGGCCTTTCTGCGTTTATA |

**Supplementary References:**

1. Meyer, A.J., Segall-Shapiro, T.H. and Voigt, C.A. (2018) Marionette: *E. coli* containing 12 highly-optimized small molecule sensors. *bioRxiv*.
